# Incidence, prevalence and clinical presentation of inflammatory bowel diseases in Northern France: a 30-year population-based study

**DOI:** 10.1016/j.lanepe.2024.101097

**Published:** 2024-10-18

**Authors:** Hélène Sarter, Thibaut Crétin, Guillaume Savoye, Mathurin Fumery, Ariane Leroyer, Luc Dauchet, Thierry Paupard, Hugues Coevoet, Pauline Wils, Nicolas Richard, Dominique Turck, Delphine Ley, Corinne Gower-Rousseau, EPIMAD study Group, Eric Agoute, Najib Al Ghossaini, Raied Al Hameedi, Myriam Al Khatib, Saria Al Turk, Jean-Marie Andre, Matthieu Antoine, Michel Antonietti, Amar Aouakli, Laura Armengol-Debeir, Ibrahim Aroichane, Fadi Assi, Eric Auxenfants, Alina Avram, Kassem Azzouzi, Damyan Bankovski, Bernard Barbry, Nicolas Bardoux, Philippe Baron, Anne Baudet, Pauline Bayart, Brice Bazin, Arash Bebahani, Jean-Pierre Becqwort, Houssem Ben Ali, Emmanuel Ben Soussan, Coralie Benard, Vincent Benet, Corinne Benguigui, Abdeslam Bental, Sara Bentaleb-Bellati, Isabelle Berkelmans, Jacques Bernet, Karine Bernou, Nathalie Bertiaux-Vandaele, Pauline Bertot, Valérie Bertrand, Emilie Biloud, Nathalie Biron, Benjamin Bismuth, Cyril Blanchard, Maurice Bleuez, Fabienne Blondel, Valérie Blondin, Marius Bobula, Philippe Bohont, Eléonore Boivin, Vanessa Bon Djemah, Eric Boniface, Philippe Bonniere, Pierre Bonvarlet, Arnaud Boruchowicz, Raoul Bostvironnois, Médina Boualit, Ahlem Bouazza, Bruno Bouche, Christian Boudailler, Claude Bourgeaux, Morgane Bourgeois-Fumery, Arnaud Bourguet, Agnès Bourienne, Hamza Boutaleb, Alexis Bouthors, Julien Branche, Franck Brazier, Marie Bridenne, Hélène Brihier, Laura Bril, Philippe Bulois, Pierre Burgiere, Joël Butel, Jean-Yves Canva, Valérie Canva-Delcambre, Florence Cardot, Sandrine Carette, Pierre Carpentier, Michel Cassagnou, Jean-François Cassar, François Castex, Pascale Catala, Stéphane Cattan, Sylviane Catteau, Bernard Caujolle, Gérard Cayron, Catherine Chandelier, Cloé Charpentier, Marthe Chavance-Thelu, Agathe Cheny, Dinu Chirita, Antoine Choteau, Jean-François Claerbout, Pierre-Yves Clergue, Hugues Coevoet, Gil Cohen, Marie Colin, Régis Collet, Jean-Frédéric Colombel, Stéphanie Coopman, Lucie Cordiez, Antoine Cortot, Jean Corvisart, Frédéric Couttenier, Jean-François Crinquette, Valérie Crombe, Abdelhakim Daoudi, Vincent Dapvril, Thierry Davion, Sébastien Decoster, Laurent Defontaine, Nicolas Degrave, Aurélien Dejaeger, Richard Delcenserie, Marine Deleplanque, Dorothée Delesalle, Olivier Delette, Thierry Delgrange, Laurence Delhoustal, Jean-Stéphane Delmotte, Sabri Demmane, Guy Deregnaucourt, Constance Deschepper, Jean-Pierre Desechalliers, Patrick Desmet, Pierre Desreumaux, Gérard Desseaux, Philippe Desurmont, Alain Devienne, Eve Devouge, Alex Devroux, Arnaud Dewailly, Sébastien Dharancy, Aude Di Fiore, Emmanuel Diaz, Djamal-Dine Djeddi, Rachid Djedir, Wissam Doleh, Marie-Laure Dreher-Duwat, Richard Dubois, Clothilde Duburque, Frédéric Ducrot, Philippe Ducrotte, André Dufilho, Christian Duhamel, Caroline Dumant-Forest, Jean-Louis Dupas, Frédéric Dupont, Yves Duranton, Arnaud Duriez, Nicolas Duveau, Mohammadi El Farisi, Khalil El Hachkar, Caroline Elie, Marie-Claire Elie-Legrand, Matthieu Eoche, Essmaeel Essmaeel, Dominique Evrard, Jean-Paul Evrard, Armelle Fatome, Karima Fellah-Sekkai, Bernard Filoche, Laurent Finet, Mathilde Flahaut, Camille Flamme, David Foissey, Peggy Fournier, Philippe Foutrein, Marie-Christine Foutrein-Comes, Thierry Frere, Mathurin Fumery, Julie Galand, Philippe Gallais, Claudine Gamblin, Serge Ganga, Romain Gerard, Guillaume Geslin, Yves Gheyssens, Salah Ghrib, Thierry Gilbert, Bénédicte Gillet, Denis Godart, Jean-Michel Godchaux, Guetty Goeguebeur, Odile Goria, Frédéric Gottrand, Philippe Gower, Lucien Grados, Brigitte Grandmaison, Marion Groux, Claire Guedon, Loïc Guerbeau, Mathilde Gueroult-Dero, Jean-François Guillard, Laurence Guillem, François Guillemot, Dominique Guimber, Baya Haddouche, Vincent Hautefeuille, Philippe Hecketsweiller, Geneviève Hecquet, Jean-Pierre Hedde, Hassina Hellal, Pierre-Emmanuel Henneresse, Michel Heraud, Sophie Herve, Bruno Heyman, Patrick Hochain, Philippe Houcke, Lucie Houssin-Baillly, Bruno Huguenin, Silviu Iobagiu, Shata Istanboli, Alexsandar Ivanovic, Isabelle Iwanicki-Caron, Eric Janicki, Marine Jarry, Charlotte Jean Bart, Claude Jonas, Julia Jougon, Anne Jouvenet, Naeim Kassar, Fadi Katherin, Alfred Kerleveo, Ali Khachfe, Alfred Kiriakos, Jean Kiriakos, Olivier Klein, Matthieu Kohut, Richard Kornhauser, Demetrios Koutsomanis, Jean-Eric Laberenne, Eric Lacotte, Guy Laffineur, Marine Lagarde, Anouck Lahaye, Arnaud Lalanne, Ambroise Lalieu, Pierre Lannoy, José Lapchin, Michel Laprand, Denis Laude, Christian Le Couteulx, Charles Le Goffic, Alain Le Grix, Jean-Philippe Le Mouel, Pauline Le Roy, Rachida Leblanc, Paul Lecieux, Stéphane Lecleire, Nathalie Leclerc, Jean Ledent, Jean Lefebvre, Pascale Lefilliatre, Céline Legrand, Patrick Lelong, Bernard Leluyer, Caroline Lemaitre, Lucie Lepileur, Antoine Leplat, Elodie Lepoutre-Dujardin, Gabriel Leppeut, Henri Leroi, Maryvonne Leroy, Benoît Lesage, Jocelyn Lesage, Xavier Lesage, Isabelle Lescanne-Darchis, Dominique Lescut, Bruno Leurent, Delphine Ley, Michel Lhermie, Louise Libier, Bernard Lisambert, Isabelle Loge, Julien Loreau, Alexandre Louvet, Joséphine Lozinguez, Henri Lubrez, Damien Lucidarme, Jean-Jacques Lugand, Olivier Macaigne, Denis Maetz, Dominique Maillard, Hubert Mancheron, Olivia Manolache, Anne-Bérengère Marks-Brunel, Charline Marre, Raymond Marti, Eric Marzloff, Philippe Mathurin, Jacques Mauillon, Vincent Maunoury, Jean-Luc Maupas, Michèle-Ange Medam Djomo, Chloé Melchior, Ziad Melki, B. Mesnard, Patrice Metayer, Lofti Methari, Franck Meurisse, Laurent Michaud, Patricia Modaine, Angélique Monthe, Loïk Morel, Mathilde Morin, Pierre-Eugène Mortier, Perrine Mortreux, Olivier Mouterde, Nicolas Mozziconaci, Jean Mudry, Maria Nachuri, Minh Dung Ngo, Eric N'guyen Khac, Bertrand Notteghem, Vincent Ollevier, Atika Ouraghi, Barriza Oussadou, Dominique Ouvry, Bernard Paillot, Claire Painchart, Nicole Panien-Claudot, Christian Paoletti, Arsène Papazian, Bruno Parent, Jean-Claude Paris, Philippe Patrier, Thierry Paupard, Bernard Pauwels, Mathieu Pauwels, Richard Petit, Muriel Piat, Sandrine Piotte, Christophe Plane, Bernard Plouvier, Eric Pollet, Pierre Pommelet, Daniela Pop, Charlotte Pordes, Gérard Pouchain, Philippe Prades, Jean-Christophe Prevost, Manon Pruit, Gilles Quartier, Anne-Marie Queuniet, Jean-François Quinton, Alain Rabache, Gilles Raclot, Sébastien Ratajczyk, Nicole Reix, Thibaud Renaut-Vantroys, Marine Revillion, Ghassan Riachi, Clémentine Riault, Nicolas Richard, Cécile Richez, Benoît Rimbert, Philippe Robinson, Juan Daniel Rodriguez, Jean Roger, Jean-Marc Roux, Alain Rudelli, Clémence Saingier, Guillaume Savoye, Patrick Schlossberg, David Sefrioui, Michel Segrestin, David Seguy, Célik Seminur, François Sevenet, Jean Silvie, Claire Spyckerelle, Nathalie Talbodec, Noémie Tavernier, Henriette Tchandeu, Aurore Techy, Jean-Luc Thelu, Henri Thiebault, Jean-Marie Thorel, Christophe Thuillier, Guillaume Tielman, Manuella Tode, Jean Tonnel, Jean-Yves Touchais, Audrey Toulemonde-Huguet, Pierre Toumelin, Yvan Touze, Léa Tran, Jean-Luc Tranvouez, Nadia Triki, Dominique Turck, Justine Turpin, Eric Vaillant, Claude Valmage, Dominique Vanco, Nathalie Vandaele-Bertiaux, Hélène Vandamme, Elise Vander Eeken, Etienne Vanderbercq, Philippe Vandermollen, Philippe Vandevenne, Lionel Vandeville, Alain Vandewalle, Jean-Pierre Vanhoove, Audrey Vanrenterghem, Charlotte Vanveuren, Iona Vasies, Guy Verbiese, Juliette Verlynde, Philippe Vermelle, Christine Verne, Gwenola Vernier-Massouille, Perrine Vezelier-Cocq, Juliette Viart, Benoît Vigneron, Marc Vincendet, Jacques Viot, Y.M. Voiment, Jean-Yves Wallez, Michel Wantier, Faustine Wartel, Jean-Christian Weber, Jean-Louis Willocquet, Nathalie Wizla, Eric Wolschies, Tajiogue Yimfor, Oana Zahara, Alberto Zalar, Sonia Zaoui, Anne Zellweger

**Affiliations:** a CHU Lille, Public Health, Epidemiology and Economic Health Unit, EPIMAD Registry, Maison Régionale de la Recherche Clinique, F-59000 Lille, France; b Univ. Lille, Inserm, CHU Lille, U1286 - INFINITE - Institute for Translational Research in Inflammation, Lille F-59000, France; c Gastroenterology Unit, CHU Lille, University of Lille, Lille F-59000, France; d Gastroenterology Unit, Saint Philibert Hospital, Catholic University, Lille, France; e Univ Rouen Normandie, INSERM, ADEN UMR1073, “Nutrition, Inflammation and Microbiota-gut-brain axis”, CHU Rouen, Department of Gastroenterology, Rouen F-76000, France; f Gastroenterology Unit, Amiens University Hospital, and Peritox, UMRI01, Université de Picardie Jules Verne, Amiens, France; g Univ. Lille, INSERM, CHU Lille, Institut Pasteur de Lille, U1167 - RID-AGE - Facteurs de Risque et Déterminants Moléculaires des Maladies liées au Vieillissement, Lille F-59000, France; h Gastroenterology Unit, Dunkerque Hospital, France; i Gastroenterology Unit, Les Bonnettes Private Hospital, Arras, France; j CHU Lille, Division of Gastroenterology, Hepatology, and Nutrition, Department of Paediatrics, Lille F-59000, France; k Research and Public Health Unit, Robert Debré Hospital, Reims University Hospital, France

**Keywords:** Inflammatory bowel disease, Incidence, Prevalence, Population-based registry, Crohn’s disease, Ulcerative colitis

## Abstract

**Background:**

In industrialized countries, the incidence of inflammatory bowel disease (IBD) appears stabilized. This study examined the incidence and phenotype of IBD in Northern France over a 30-year period.

**Methods:**

Including all IBD patients recorded in the EPIMAD population-based registry from 1988 to 2017 in Northern France, we described the incidence and clinical presentation of IBD according to age, sex and time.

**Findings:**

A total of 22,879 incident IBD cases were documented (59% (n = 13,445) of Crohn’s disease (CD), 38% (n = 8803) of ulcerative colitis (UC), 3% (n = 631) of IBD unclassified (IBDU)). Over the study period, incidence of IBD, CD and UC was 12.7, 7.2 and 5.1 per 10^5^ person-years, respectively. The incidence of CD increased from 5.1/10^5^ in 1988–1990 to 7.9/10^5^ in 2015–2017 (annual percent change (APC): +1.9%, p < 0.0001). The incidence of UC increased from 4.5/10^5^ to 6.1/10^5^ (APC: +1.3%, p < 0.0001). The largest increase was observed in children (+4.3% in CD, p < 0.0001; +5.4% in UC, p < 0.0001) followed by young adults aged 17–39 years (+1.9% in CD, p < 0.0001; +1.5% in UC, p < 0.0001). The increase in UC incidence was significantly higher in women than in men (+1.9% in women, +0.8% in men; p = 0.006). We estimated that in our area, by 2030, nearly 0.6% of the population will have IBD.

**Interpretation:**

The persistent increase of IBD incidence among children and young adults but also in women with UC in Northern France, suggests the persistence of substantial predisposing environmental factors.

**Funding:**

Santé Publique France; INSERM; Amiens, Lille and Rouen University Hospitals.

**Research in context**

**Evidence before this study**

We searched PubMed for research articles published from database inception until Dec 31st 2022, with no language restrictions, using the terms “Inflammatory Bowel Disease”, “Incidence” and “epidemiology”. In industrialized countries, the incidence of inflammatory bowel disease (IBD) appears to have stabilized or even declined in some countries. However, prospective population-based studies with expert-reviewed information on the patients’ diagnoses and disease characteristics are lacking to confirm this change.

**Added value of this study**

In a large population-based study performed over a 30-year period, we showed that the incidence of Crohn’s disease (CD) and ulcerative colitis (UC) is still rising in Northern France, particularly in children and young adults. In UC, incidence is rising more sharply in women and is nowadays reaching that in men. We project that by 2030, nearly 0.6% of the whole population in this French area will suffer from IBD (+30% in 10 years).

**Implications of all the available evidence**

These findings underscore i) the need for healthcare systems to prepare to face the increase of IBD patients, especially in the elderly, in the future ii) the need to a deeper understanding of environmental risk factors for IBD that particularly affect children and young people, but also women in UC.

## Introduction

Inflammatory bowel disease (IBD) encompasses chronic disorders that cause inflammation of the gastro-intestinal tract, Crohn’s disease (CD), ulcerative colitis (UC) and IBD unclassified (IBDU).^1^^,^^2^ Over the past decades, IBD has emerged as a public health challenge worldwide.^3^ It is estimated that about 1.3 million people suffer from IBD in Europe, corresponding to 0.2% of the population.^4^ Although IBD was initially considered as a Western disease, its epidemiology is changing.^5^^,^^6^ A recent systematic review of population-based studies (119 studies) revealed that overall incidence rates of IBD have stabilized in Western countries since 1990.^7^ However, incidence rates are still rising in some western countries and in children.^8^, ^9^, ^10^, ^11^, ^12^ Another concern is the increased prevalence of IBD, especially in the elderly.^13^

Since 1988, a large prospective, population-based registry (EPIMAD) on IBD has been built in Northern France, enabling to study the incidence of IBD and its changes over time.^11^^,^^14^, ^15^, ^16^

The objectives of the present study were to assess the incidence and clinical presentation of IBD in the general population over a 30-year period, to assess temporal trends in incidence according to age and sex and to estimate prevalence in 2030.

## Methods

### The EPIMAD registry

All patients with IBD recorded in the EPIMAD registry in Northern France from 1988 to 2017 were included.

The EPIMAD registry covered, in 2017, 5,899,200 inhabitants corresponding to 9% of the total French population. The EPIMAD registry’s methodology has been described in detail elsewhere.^14^^,^^15^ Briefly, eight interviewer practitioners collect data from medical chart on all incident IBD patients diagnosed by all gastroenterologists (n = 265) from private and public sector, using a standardized questionnaire. The gastroenterologist reports to the EPIMAD registry every patient consulting for the first time with symptoms suggestive of IBD. For each new incident case, an interviewer practitioner visits the gastroenterologist’s office and collects the data.

### Data collection

The main data recorded are age, sex, date of diagnosis, the time interval between symptoms onset and diagnosis, the clinical presentation, and the radiological, endoscopic and histological findings at the time of diagnosis. A diagnosis of CD, UC or IBDU is established by two expert gastroenterologists.^14^ Inter-expert concordance rate was 98% in 2018. Discrepancies are resolved through discussion between the two experts, with involvement of a third expert if necessary. The anatomic sites and CD behavior are defined according to the Montreal Classification.^17^ Data on age, sex, date of diagnosis are exhaustive. The time interval between symptoms onset and diagnosis is known in 94% of cases and missing data were removed for the description of this variable. We have access to all radiological and endoscopic findings at diagnosis. When no sufficient data was available at time of initial management by the gastroenterologist, the patient is classified as possible case and followed every 2 years for a period of 10 years. A patient classified as definite or probable IBD and included in this study had sufficient data to assess the diagnosis. For upper gastrointestinal involvement, we made the hypothesis that patients without fibroscopy do not have an upper gastrointestinal involvement that would have led to a fibroscopy in case of symptoms.

### Statistical analysis

Distribution of continuous variables was checked graphically and using normality tests. Continuous variables were expressed as the median (interquartile range [IQR]), categorical variables as the frequency (percentage). Intergroup comparisons were performed using the Wilcoxon-Mann-Whitney test for continuous variables and the chi-squared or Fisher’s exact tests for categorical variables. Changes over time in categorical variables were assessed using a chi-square test for trend, when appropriate.

Incidence rates were computed as the number of incident cases divided by the population at risk. The incidence rates were standardized by age with weightings for the European population^18^ and determined for the population as a whole, for each age group (<17, 17–39, 40–59, and ≥60 years), by sex and for ten consecutive 3-year periods. 95% confidence interval (CI) were based on a gamma distribution.^19^ Yearly population data by age-group and sex were obtained from census data from the INSEE (National Institute of Statistics and Economic Studies). A focus on incidence rates according to age was performed in pediatric-onset patients (<17 years). For this analysis we used the threshold for very-early-onset IBD^20^ and the threshold from the Paris classification^21^ leading to the following three age groups: 0–5 years, 6–9 years and ≥10 years. Incidence rates were not studied according to sex in these age groups due to the very low number of patients when stratifying according to age and sex.

Differences in incidence according to age, sex or time were tested using log-linear Poisson regressions that took account of the number of person-years at risk (introduced as an offset variable) and over-dispersion, when necessary. Differences according to age and sex were presented as Incidence Rate Ratio (IRR) by exponentiation of the coefficients for age or sex. To assess linear time trends, the calendar year was introduced as a predictor variable. Time trends were presented as the annual percent change (APC) estimated by exponentiation of the time coefficient from the Poisson regression estimates. To assess differences in time trends according to sex or age, interactions year∗age and year∗sex were included in the model.

To estimate the prevalence of IBD: i) we considered a linear increase of IBD cases in our area from 1975 to 1987 with the same distribution according to type of IBD, age and sex than in 1988–1990; ii) between 1988 and 2017, incident cases were those observed in EPIMAD registry; iii) the annual incidence rates from 2018 to 2030 were projected from the APCs observed in 1988–2017 for each age group, sex, and type of IBD and were applied to population projection provided by the INSEE.^22^ An aging process was then applied to each incident case from diagnosis year to 2030 using yearly survival rates by age, sex and birth cohort provided by the INSEE.^23^ Prevalence rates were computed with their 95% confidence intervals. Prevalence in 2030 was also assessed using the lower and upper bounds of the APCs.

The statistical analysis was performed using SAS software (v9.4, SAS Institute Inc., Cary, NC, USA) and R software v3.6.1 (R Foundation for Statistical Computing, Vienna, Austria). The threshold for statistical significance was set to p ≤ 0.05.

The study protocol has been approved by the French Ministry of Health according to the regulation of the registries in general population (Number n°97.107 for Advisory Committee on the Processing of Health Research Information (CCTIRS) and 917,089 for the French Data Protection Authority (CNIL)).

### Role of the funding source

The funders of the study had no role in the study design, data collection, data analysis, data interpretation, or writing of the manuscript.

## Results

### Incidence of IBD

Between January 1st, 1988, and December 31st, 2017, we identified 22,879 incident cases of IBD, including 13,445 cases of CD (59%), 8803 cases of UC (38%), and 631 cases of IBDU (3%). During the study period, the age-standardized incidence of IBD was 12.7/10^5^ person-years (95% CI: [12.5–12.8]), and the incidence of CD, UC, and IBDU was respectively 7.2/10^5^ [7.1–7.3], 5.1/10^5^ [5.0–5.2], and 0.37/10^5^ [0.34–0.40].

### Clinical presentation at IBD diagnosis

The median [IQR] age at diagnosis was 26 [20–38] years for CD and 35 [25–48] years for UC (Table 1). Ten per cent of patients (n = 2329) had a family history of IBD. Median time interval between symptoms onset and diagnosis was 3 months [1–9] in CD and 2 months [1–6] in UC (p < 0.0001) without change over time. Extra-intestinal manifestations were present in 8% (n = 1899) of the patients.

**Table 1:** Sociodemographic and clinical data of IBD patients from a prospective population-based registry in Northern France from 1988 to 2017 (n = 22,879).

Characteristics at diagnosis	All patients	Males	Females	p-value
	(n = 22,879)	(n = 10,925)	(n = 11,954)	
**Disease type**				
CD	13,445 (58.8%)	5910 (54.0%)	7535 (63.0%)	<0.0001
UC	8803 (38.5%)	4699 (43.0%)	4104 (34.3%)	
IBDU	631 (2.8%)	316 (3.0%)	315 (2.6%)	
**CD**				
Median [IQR] age, years	26 [20–38]	26 [19–39]	26 [20–38]	0.128
Female gender	7535 (56.0%)			
Family history of IBD	1700 (12.6%)	720 (12.2%)	980 (13.0%)	0.154
Median [IQR] time between symptoms onset and diagnosis, months	3 [1; 9]	3 [1; 8]	3 [1; 9]	0.001
Disease site^a^				
L1	2666 (20.4%)	1176 (20.6%)	1490 (20.3%)	0.476
L2	3913 (30.0%)	1681 (29.4%)	2232 (30.4%)	
L3	6467 (49.6%)	2853 (50.0%)	3614 (49.3%)	
L4	2951 (21.9%)	1418 (24.0%)	1533 (20.3%)	<0.0001
Behavior^a^^,^^b^				
B1	3840 (73.8%)	1443 (73.7%)	1769 (74.9%)	0.550
B2	928 (17.8%)	357 (18.2%)	420 (17.8%)	
B3	433 (8.3%)	158 (8.1%)	172 (7.3%)	
Perianal disease	658 (4.9%)	374 (6.3%)	284 (3.8%)	<0.0001
Extra-intestinal manifestations	1565 (11.6%)	647 (10.9%)	918 (12.2%)	0.027
**UC**				
Median [IQR] age, years	35 [25–48]	38 [27–51]	32 [24–44]	<0.0001
Female gender	4104 (46.6%)			
IBD family history	594 (6.7%)	264 (5.6%)	330 (8.0%)	<0.0001
Median [IQR] time between symptoms onset and diagnosis, months	2 [1; 6]	2 [1; 6]	2 [1; 6]	0.013
Disease site^a^				
E1	3157 (36.3%)	1536 (33.1%)	1621 (40.0%)	<0.0001
E2	3210 (36.9%)	1802 (38.8%)	1408 (34.7%)	
E3	2329 (26.8%)	1304 (28.1%)	1025 (25.3%)	
Extra-intestinal manifestations	299 (3.4%)	138 (2.9%)	161 (3.9%)	0.011

a According to the Montreal classification.

b Recorded in the EPIMAD registry since 2008.

In CD, 50% of the patients (n = 6467) had ileocolonic (L3) involvement, 30% (n = 3913) had pure colonic (L2) disease, and 20% (n = 2666) had pure ileal (L1) disease; 22% (n = 2951) displayed upper gastrointestinal involvement (L4). Five percent of the patients with CD (n = 658) had perianal abscesses and/or fistulae at diagnosis. At diagnosis, 74% of CD patients presented an inflammatory behavior (n = 3840), 18% a stricturing behavior (n = 928), and 8% a penetrating behavior (n = 433).

At diagnosis, UC was classified as proctitis (E1) in 36% (n = 2741) of the patients, left-sided colitis (E2) in 35% (n = 2640), and extensive colitis (E3) in 29% (n = 2257).

### Incidence and clinical presentation by sex

Results of sociodemographic and clinical data by sex are detailed in Table 1.

The incidence rate of CD was significantly higher in women (8.0/10^5^ [7.8–8.2]) than in men (6.4 [6.3–6.6]) corresponding to an Incidence Rate Ratio (IRR) of 1.25 [1.19–1.31] (p < 0.0001, Supplementary Table S1). Median age at diagnosis did not differ according to sex (p = 0.128).

The incidence rate of UC was significantly lower in women (4.5 [4.4–4.7]) than in men (5.7 [5.5–5.9]), corresponding to an IRR of 0.83 [0.78–0.89] (p < 0.0001); median age at UC diagnosis was significantly lower in women than in men (32 [24–44] versus 38 [27–51] years respectively; p < 0.0001).

### Incidence and clinical presentation by age group

**Fig. 1:**
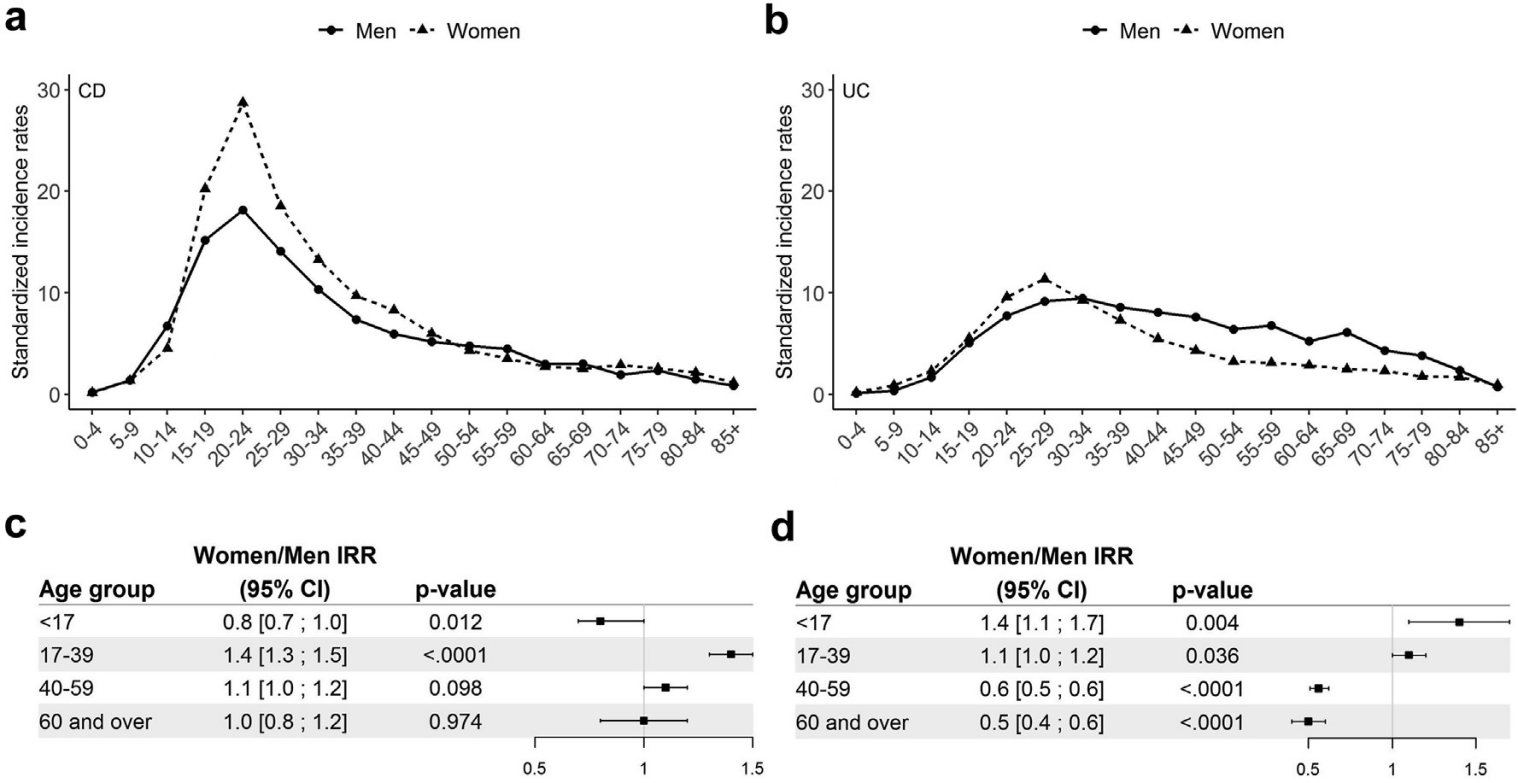
Incidences rates of CD (n = 13,445, panel a) and UC (n = 8,803, panel b) in Northern France over the study period (1988–2017), by sex and 5-year age groups; Women/Men incidence rate ratio (IRR) according to age group in CD (panel c) and UC (panel d). Incidence rates with confidence intervals are given in Supplementary Table S6.

Sociodemographic and clinical data are summarized by age group in Supplementary Table S2. The 1988–2017 incidence rate of CD was significantly higher in the 17–39 years age-group (15.4/10^5^ [15.1–15.8]) than in the <17 years (3.7 [3.5–3.9]), the 40–59 years (5.3 [5.1–5.6]) and the ≥60 years age-groups (2.4 [2.3–2.6]) (Supplementary Table S1, p < 0.0001). Likewise, in UC, the incidence rate was significantly higher in the 17–39 years age-group (8.7 [8.4–8.9]) than in the <17 years (1.4 [1.3–1.5], the 40–59 years (5.6 [5.4–5.8]) and the ≥60 years age-groups (3.1 [2.9–3.3]; p < 0.0001). As shown in Fig. 1, there was a significant interaction between sex and age in both CD (p < 0.0001) and UC (p < 0.0001). In CD, incidence was significantly higher in men than in women in the <17 years age-group (IRR: 0.8 [0.7–1.0], p = 0.012), whereas significantly more women were observed in the 17–39 years age-group (IRR: 1.4 [1.3–1.5], p < 0.0001). In UC, an inversion of the sex ratio was also observed with age; incidence was significantly higher in women in the <17 and 17–39 years age-groups (IRR: 1.4 [1.1–1.7], p = 0.004 and 1.1 [1.1–1.2], p = 0.036 respectively) and significantly higher in men in the 40–59 and ≥ 60 years age-groups (IRR: 0.6 [0.5–0.6], p < 0.0001 and 0.5 [0.4–0.6], p < 0.0001 respectively).

### Temporal trends of incidences

**Fig. 2:**
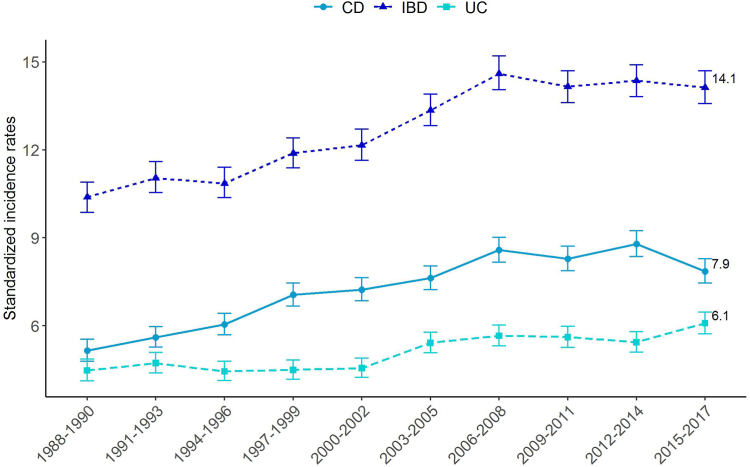
Changes over time in standardized incidence rates for IBD (n = 22,879), CD (n = 13,445) and UC (n = 8803) in Northern France from 1988 to 2017.

The overall incidence of IBD increased from 10.4 [9.9–10.9]/10^5^ person-years in 1988–1990 to 14.1/10^5^ [13.6–14.7] in 2015–2017 (Annual Percent Change, APC: +1.5% [1.2–1.8]; p < 0.0001). The incidence of CD increased from 5.1 [4.8–5.5] to 7.9 [7.4–8.3] (APC: +1.9% [1.6–2.2]; p < 0.0001), and the incidence of UC also significantly increased from 4.5 [4.1–4.9] to 6.1 [5.7–6.5] (APC: +1.3% [0.9–1.7]; p < 0.0001) (Fig. 2).

#### Inflammatory bowel disease overall

The increase in IBD incidence did not significantly differ according to sex (time∗sex interaction p = 0.229). On contrast, time trends significantly differed according to age (time∗age interaction p < 0.0001), with stability in the ≥60 years age-group (APC: −0.5% [−1.1–0.1], p = 0.131) and the highest rise observed in the <17 years age-group (APC: +4.6% [3.9–5.2], p < 0.0001) (Supplementary Table S3).

#### Crohn’s disease

**Fig. 3:**
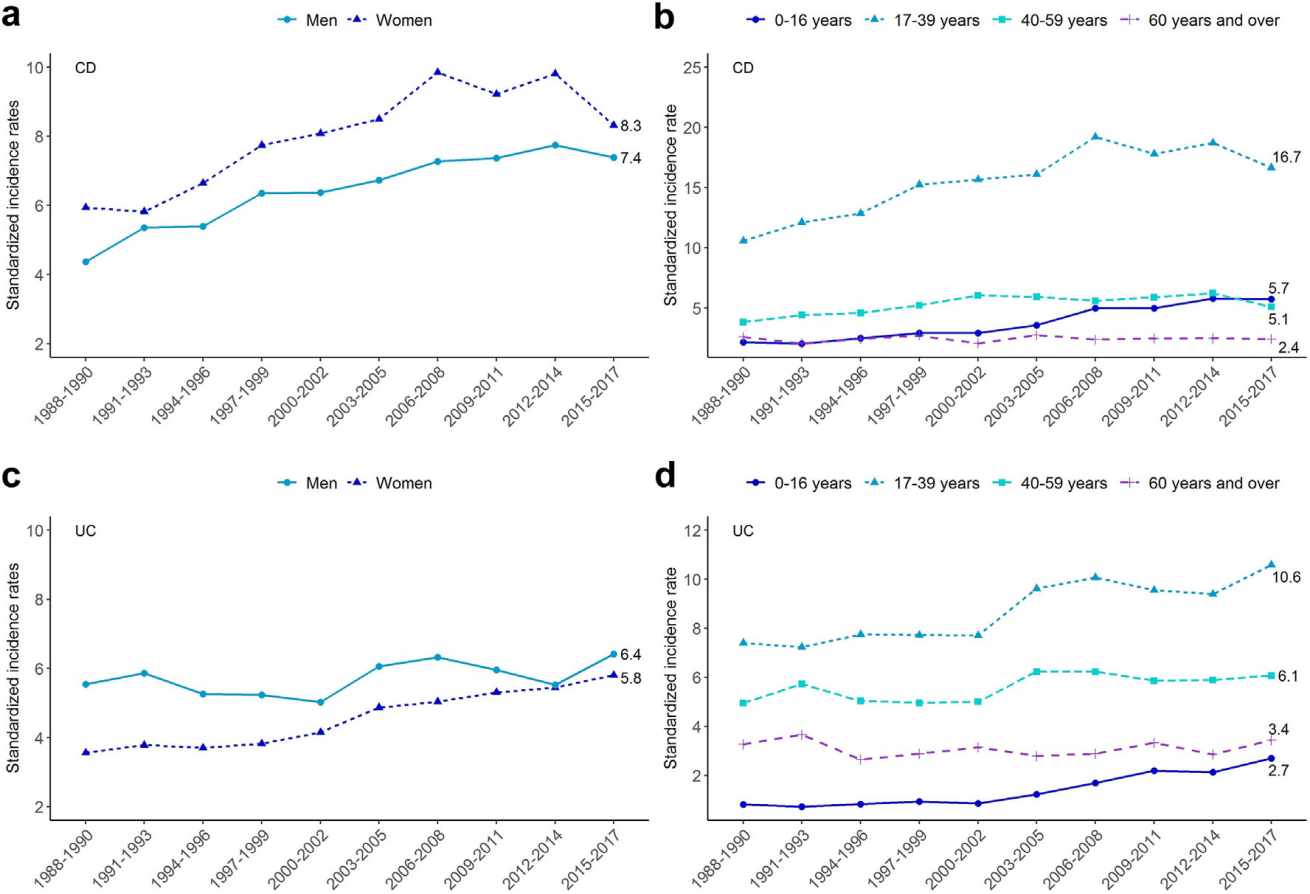
Changes over time in the standardized incidence rates for CD (n = 13,445) and UC n = 8803) in Northern France from 1988 to 2017 by sex and age group. a) CD incidence according to sex. b) CD incidence according to age. c) UC incidence according to sex. d) UC incidence according to age. Incidence rates with confidence intervals are given in Supplementary Table S7.

**Fig. 4:**
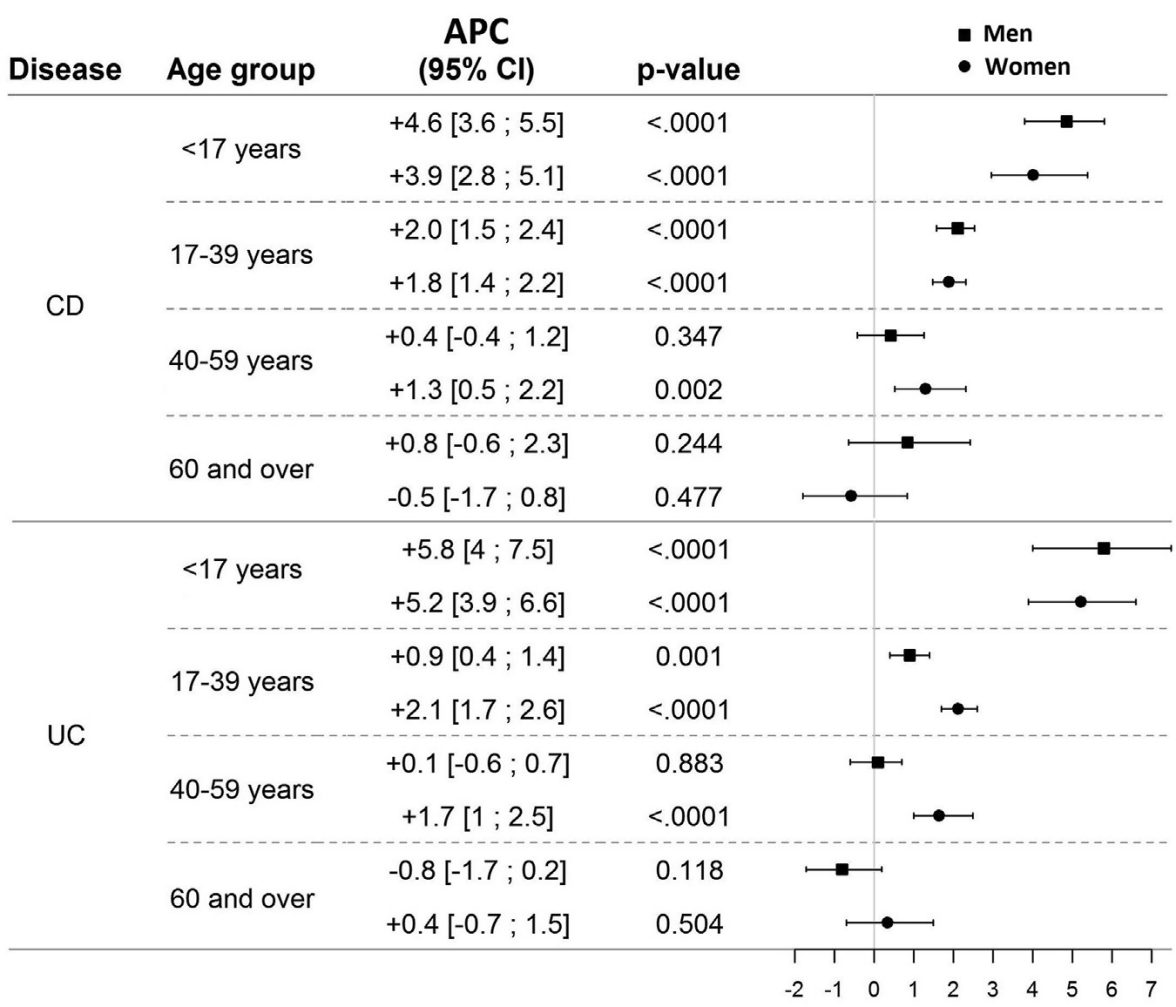
Annual percent change (APC) in %/year over the 1988–2017 period in patients with CD (n = 13,445) and patients with UC (n = 8803) in Northern France by age group and sex.

The time trends in CD did not differ according to sex (time∗sex interaction: p = 0.365) (Fig. 3A). Among patients with CD, the sex ratio (F/M) was stable over time (p = 0.085), fluctuating between 1.1 and 1.4. On contrary, time trends differed according to age (time∗age interaction: p < 0.0001) (Fig. 3B). The highest increase was observed in the <17 years age-group (APC: +4.3% [3.5–5.1], p < 0.0001), followed by the 17–39 years age-group (APC: +1.9% [1.5–2.2], p < 0.0001), and the 50–59 years age-group (APC: +0.9% [0.3–1.5], p = 0.006). In the ≥60 years age-group CD incidence remained stable over time (APC: +0.1% [−1.0–1.1], p = 0.893). In each age group, time trends were not statistically different between men and women (time∗sex interaction: p = 0.387, 0.661, 0.071 and 0.101 in <17, 17–39, 40–59 and ≥ 60 years age groups, respectively) (Fig. 4, Supplementary Fig. 1A and B and Table S4).

#### Ulcerative colitis

In UC, time trends differed significantly according to sex (interaction: p = 0.006). The increase in UC incidence was significantly higher in women (APC: +1.9% [1.3–2.6]; p < 0.0001 versus +0.8% [0.2–1.3]; p = 0.006) with incidence in women being close to that of men at the end of study period (Fig. 3C). The female/male ratio rose over time, from 0.7 in 1988–1990 to 0.9 in 2015–2017 (trend test: p < 0.0001). Time trends in UC significantly differed according to age (p < 0.0001, Fig. 3D) but also according to age and sex. In <17 years age-group, time trends were not different according to sex (time∗sex interaction: p = 0.591) with APC of +5.8% [4.0–7.5] in men (p < 0.0001) and +5.2% [3.9–6.6] in women (p < 0.0001). We observed significantly different time trends by sex in the 17–39 years (interaction: p < 0.001) and 40–59 years age-groups (interaction: p = 0.003). The incidence rates increased significantly in women aged 17–39 years and 40–59 years (APC: +2.1% [1.7–2.6], p < 0.0001, and 1.7% [1.0–2.5], p=<0.0001, respectively). In men, incidence rates increased in the 17–39 years age-group by +0.9% [0.4–1.4] (p = 0.001) and were stable in 40–59 years age-group (+0.1% [−0.6–0.7], p = 0.883). In the ≥60 group age-group, time trends remained stable and did not differ significantly when comparing men and women (interaction: p = 0.102) (Fig. 4, Supplementary Fig. 1C and D and Table S5).

#### Focus on pediatric-onset IBD

From 1988 to 2017, 2103 children with an IBD diagnosis before the age of 17 years were included in the EPIMAD registry (9% of all IBD cases). Among them, 57 (3% of all pediatric IBD cases) were diagnosed before the age of 6 (very-early-onset IBD), 223 between 6 and 9 years (11%) and 1823 after the age of 10 (87%). The 1988–2017 incidence rate of IBD was significantly higher in the 10–16 years age-group (10.6/10^5^ [10.1–11.1]) than in the 6–9 years (2.3 [2.0–2.6]) and the 0–5 years (0.4 [0.3–0.5]) (p < 0.0001). IBD incidence rates significantly rose in all pediatric age groups, especially in patients 6 years and over with a threefold increase. The incidence of IBD increased from 0.4 [0.0–0.7]/10^5^ person-years in 1988–1990 to 0.9/10^5^ [0.4–1.4] in 2015–2017 in 0–5 years age-group (p = 0.010), from 1.5 [0.7–2.3] to 4.1 [2.8–5.4] in 6–9 years age-group (p < 0.0001), and from 6.3 [5.0–7.5] to 17.3/10^5^ [15.3–19.3] in 10 years and over age-group (p < 0.0001) (Supplementary Fig. S2).

### Prevalence

**Fig. 5:**
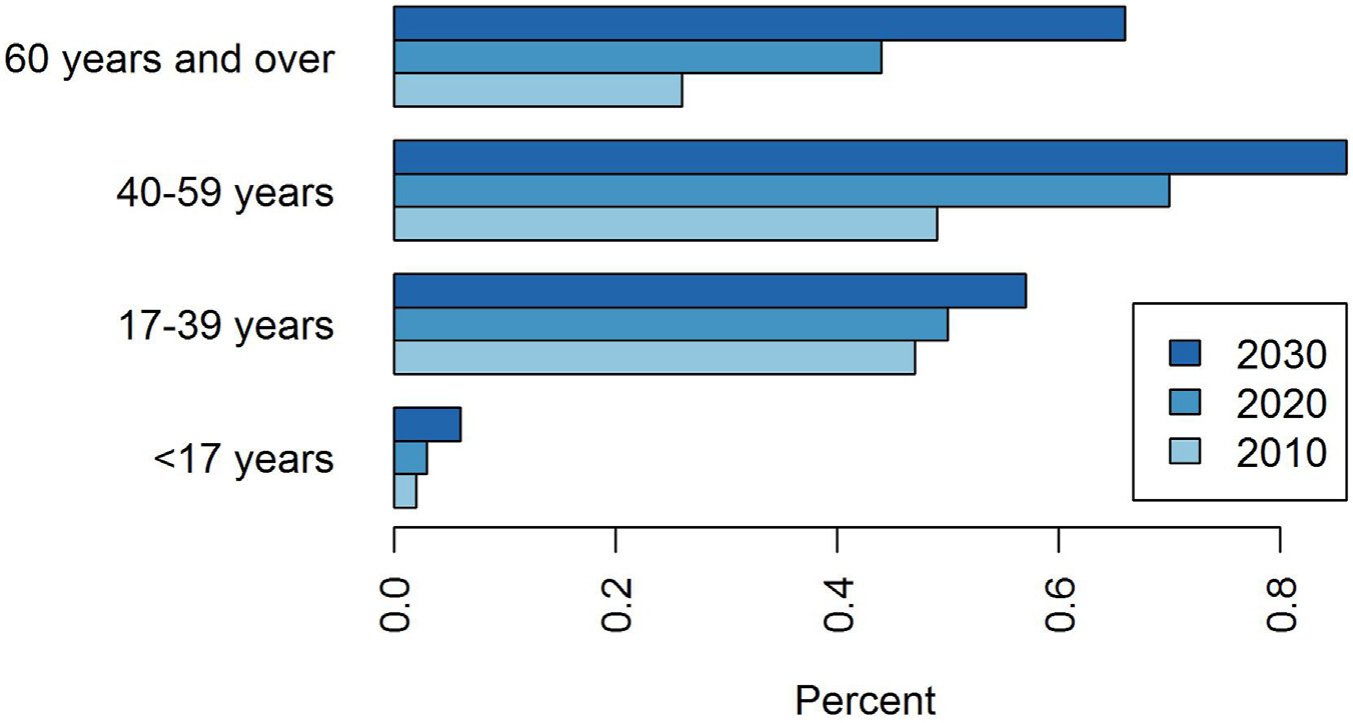
Prevalence according to age in 2010, 2020 and 2030.

Estimated prevalence was 0.31% (95% CI: [0.31–0.32]) of total population in 2010 and 0.43% [0.43–0.44] in 2020. By projecting the above mentioned results, the estimated prevalence would rise to 0.57% [0.56–0.57] in 2030 (i.e., a 30% increase in prevalent cases in 10 years). In 2030, by using respectively the lower and the upper bounds of confidence intervals of APCs, prevalence would be of 0.56% [0.55–0.56] and 0.58% [0.57–0.58], respectively. Notably, the rise in prevalence is also accompanied by an aging of the IBD population (Fig. 5), with the prevalence doubling in 20 years in the ≥60 years age-group while slightly rising in 17–39 years.

### Temporal evolution of disease sites

**Fig. 6:**
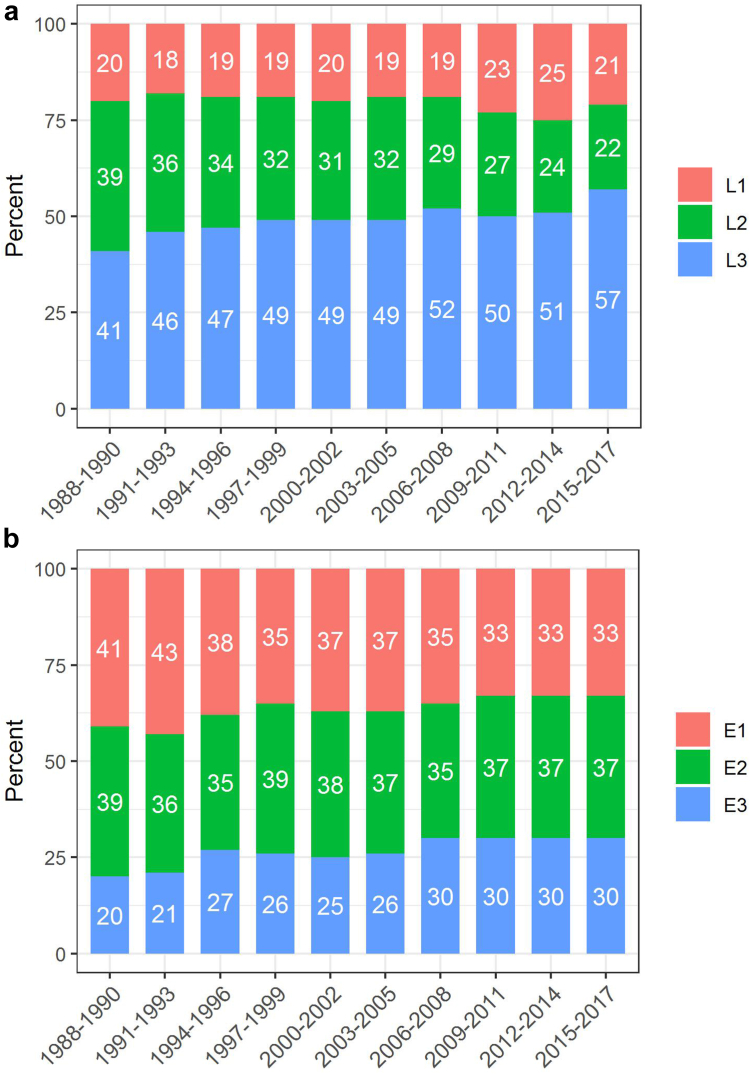
Changes in disease sites in patients with a) CD (n = 13,445) and b) UC (n = 8803) in Northern France from 1988 to 2017.

The proportion of patients with L3 disease increased significantly between 1988–1990 and 2015–2017 (from 41% to 57%), whereas the proportion with a pure colonic phenotype (L2) decreased significantly from 38% to 22% (trend p < 0.0001) (Fig. 6).

The proportion of perianal lesions remained stable over time (trend p = 0.386) as well as the distribution of disease behavior (p = 0.494).

In patients with UC, the proportion with E1 disease decreased significantly from 42% to 33%, whereas the proportion with E3 increased significantly from 20% to 30% (p < 0.0001) (Fig. 6).

## Discussion

In a large population-based registry including 22,879 incident cases of pediatric- and adult-onset IBD over a 30-year period (1988–2017), the incidence of CD rose steadily from 5.1 to 7.9 (APC: +1.9%/year) and the incidence of UC rose from 4.5 to 6.1 (APC: +1.3%). The increase in incidence was particularly marked in children and young adults. In UC, incidence also increased more sharply in women than in men. Incidence of very-early-onset (<6 years) and early-onset (6–9 years) pediatric IBD was low with incidence rates of 0.4 and 2.3, respectively as compared to 10.6 in 10 years and over. Incidence rate rose significantly in all pediatric age groups with a threefold increase in both 6–9 years and 10 years and over age-groups. Based on these results, we estimate that by 2030, about 0.6% of the adult population in Northern France will suffer from IBD, with an aging of IBD population.

In the literature, the annual incidence rates in Europe range from 0.4 to 22.8 per 10^5^ for CD, and from 2.4 to 44.0 per 10^5^ for UC.^4^ In the present study, the CD/UC ratio was above 1, contrasting with most of the other population-based studies performed in Western Europe.^4^ Since the 1990s, epidemiological studies of Western countries have shown that incidence trends have changed: more than two third of studies in CD or UC reported stable or falling incidences.^6^^,^^7^ This result contrasts with our present observation of an increase in the overall incidence of IBD in Northern France. Yet, some recent studies also highlighted a continuous rise in high incidence countries.^8^^,^^9^^,^^24^

Although the incidences appear to have stabilized in some Western countries, the overall estimated prevalence of IBD is still increasing because of a low mortality rate and aging of the IBD patients.^6^ Our estimated IBD prevalence of 0.6% in 2030 is in line with recent publications. Global prevalence of IBD in Europe is currently estimated at 0.2% with ranges from 1.5 to 331/10^5^ persons in CD and from 2.4 to 432 per 10^5^ persons in UC.^4^ Global Burden of Disease 2017 estimated the highest prevalence in USA and UK with a prevalence of about 0.45%.^25^ The results of a Scottish study with a capture-recapture design suggested that the prevalence would rise to 1% within 10 years.^26^ We report an aging of the prevalent population that was also described in the literature^26^ and has to be taken into account considering the under-representation of elderly in clinical trials and the special features of the elderly population: drug interaction and side-effects raised by polypharmacy, comorbidities and frailty, and higher IBD-related hospitalization costs.^27^

Our study’s major finding is that the trends in incidence of CD and UC are varying according to age and sex. The highest rates of increase were observed in children, followed by young adults in both CD and UC and both gender, whereas incidences were stable in 60 years old people and over. These results are in line with a recent systematic review of population-based studies that stated a significant increase in 84% of studies in children.^10^ In middle-aged patients aged 40–59 years, incidence rates were only increasing in women and especially in UC. Shah et al. described sex differences in the incidence of IBD as a function of age at diagnosis.^28^ These findings could suggest that female hormones probably play a role in IBD; we indeed observed a shift in the sex ratio at around the age of puberty and age of menopause, particularly in CD. In two large prospective American cohorts of women, the use of oral contraceptives was associated with an increased risk of CD (HR: 2.82 [1.65; 4.82] for current contraceptive use and 1.39 [1.05; 1.85] for past contraceptive use.^29^ A recent study showed that progestogen-only pills were not associated with CD.^30^ Findings from these studies support the hypothesis that the estrogenic component of contraception may contribute to the pathogenesis of IBD.

The significant rise in each pediatric age-group and especially in 6 years and over also points to the presence of environmental factors in childhood. In particular, dietary evolution with more processed food and a more and more sedentary behavior may have impacted the IBD incidence.

Current smoking has been shown to protect against UC while former smoker status was associated with an increased risk of UC–mostly in the first five years after smoking cessation.^31^ The rise in regular tobacco consumption among women since the early 1980s might contribute to the increased incidence of CD but not of UC.^32^ Overall, women usually stop smoking earlier in life than men because of pregnancy; this may play a role in the increased incidence of UC in young women.

Appendectomy is reportedly associated with a 69% lower risk of UC.^33^ The incidence of appendectomy has declined sharply since 1990, especially in women, with an inversion of the male/female sex ratio (0.85 in 1997, 1.05 in 2012).^34^ This factor may have prompted an increase in the risk of developing UC in women.

Lastly, the global increase in incidence could also be related to the development of new diagnostic tools that may have led to an earlier and more widespread diagnosis of IBD. Nevertheless, in our study, the time interval between symptoms onset and IBD diagnosis did not change over time, suggesting that diagnostic modalities remained more or less stable over the study period.

Between 1988 and 2017, the proportion of CD with ileocolonic involvement (L3) rose significantly from 41% to 57%. This could possibly be due, at least in part, to the rise in complete assessment of the bowel and to the improvement of medical investigation techniques. Yet, this trend remained when considering only patients having undergone a full bowel assessment at diagnosis or when considering only patients having undergone CT and/or MRI (sufficient data only since 2006). These variations in disease site are thus likely to reflect a real rise in ileal involvement of the disease. This can be explained, as proposed by Van Kruiningen et al., by the rising incidence in young patients since Peyer’s patches–that play a major role in intestinal immunity and that are located in the ileum–are more numerous in young adult patients than older ones.^35^ Yet, as ileal involvement is also rising in older age groups, explanations may also relate to the rise of an environmental factor that would be absorbed first in the ileum.

The present study’s major strengths were its population-based design, the large sample size, the exhaustive recording over a 30-year period using the same methodology, and the use of validated, published diagnostic criteria. The major limitation is related to the absence of data on smoking status or oral contraceptive use for the understanding of their respective role in IBD evolution. Data are also limited to those issued from medical files, with no data concerning eating behavior or other environmental variables. The major source is all the gastroenterologists and the patients are never seen by interviewer practitioners of Epimad’s registry. Therapeutic management after diagnosis is also poorly known since we recorded only data at IBD diagnosis. Disease severity is only assessed by the phenotype of the disease, without assessment by clinical activity indexes. Other data are also insufficiently available in medical files to be described like BMI and comorbidities. Ethnic origin was also unavailable since it is forbidden to record this parameter due to stringent ethical considerations in France. Moreover, data are limited to the Northern France and cannot be extrapolated to other regions in France or other countries.

In conclusion, the results of this large population-based study over a 30-year period showed that incidences of CD and UC are still rising dramatically in children, but also among young adults in Northern France. Importantly, in UC, with incidences rising more sharply in women, incidence rates for women are reaching those for men at the end of study period. These findings strongly suggest that one or more persistent major environmental factors may predispose children, young adults and women to IBD in this area. Based on our findings, we project that 0.6% of the population in Northern France will experience IBD by 2030. This underscores the importance of preparing for the increasing healthcare demands and associated costs, as well as addressing the aging of the IBD population.

## Contributors

Hélène Sarter, MS: Conceptualization, methodology, software, validation, formal analysis, data curation, visualization, writing- original draft. Thibaut Crétin, MD: Conceptualization, writing- original draft. Guillaume Savoye, MD, PhD: Conceptualization, investigation, validation, funding acquisition, writing-review and editing. Mathurin Fumery, MD, PhD: Conceptualization, validation, investigation, funding acquisition, writing-review and editing.

Ariane Leroyer, MD, PhD: Methodology, data curation, formal analysis, software, writing-review and editing. Luc Dauchet, MD, PhD: Methodology, writing-review and editing. Thierry Paupard, MD: Investigation, resources, writing-review and editing. Hugues Coevoet, MD: Investigation, resources, writing-review and editing. Pauline Wils, MD: Investigation, resources, writing-review and editing. Nicolas Richard, MD: Resources, writing-review and editing. Dominique Turck, MD: Conceptualization, investigation, validation, writing-review and editing. Delphine Ley, MD, PhD: Conceptualization, investigation, validation, writing-review and editing. Corinne Gower-Rousseau, MD, PhD: Conceptualization, investigation, funding acquisition, validation, writing-original draft. HS and CGR verified the underlying data.

## Data sharing statement

Data sharing requests will be considered by the EPIMAD study group on written request to the corresponding author. De-identified participant data and data dictionary will be available, after approval of a written proposal and a signed data access agreement.

## Declaration of interests

Guillaume Savoye has served as speaker for MSD France, Ferring France, Abbvie France, and Vifor France.

Mathurin Fumery has received lecture/consultant fees from Abbvie, Ferring, Tillots, MSD, Biogen, Amgen, Fresenius, Hospira, Pfizer, Celgene, Gilead, Boerhringer, Galapagos, Janssen and Takeda. Delphine Ley has received consultant fees from Sandoz and AbbVie. Thierry Paupard has received lecture/consultant fees from Abbvie, Amgen, Takeda, Janssen, Biogen, and Celltrion. Dominique Turck has received lecture fees from Sandoz. Nicolas Richard has received lecture/consultant fees from AbbVie and Takeda. The other authors state that they have no competing interests regarding this work to disclose.

## Appendix ASupplementary data

EPIMAD Study Group
