# Supplementary material for: Incidence, prevalence and clinical presentation of inflammatory bowel diseases in Northern France: a 30-year population-based study
Source: Lancet Reg Health Eur. 2024 Oct 18;47:101097. doi: 10.1016/j.lanepe.2024.101097 (PMC11522416; doi:10.1016/j.lanepe.2024.101097)
Supplement: EPIMAD Study Group [file mmc1.docx]

EPIMAD Study Group

| **First name** | **Last name** |
| --- | --- |
| Eric | AGOUTE |
| Najib | AL GHOSSAINI |
| Raied | AL HAMEEDI |
| Myriam | AL KHATIB |
| Saria | AL TURK |
| Jean-Marie | ANDRE |
| Matthieu | ANTOINE |
| Michel | ANTONIETTI |
| Amar | AOUAKLI |
| Laura | ARMENGOL-DEBEIR |
| Ibrahim | AROICHANE |
| Fadi | ASSI |
| Eric | AUXENFANTS |
| Alina | AVRAM |
| Kassem | AZZOUZI |
| Damyan | BANKOVSKI |
| Bernard | BARBRY |
| Nicolas | BARDOUX |
| Philippe | BARON |
| Anne | BAUDET |
| Pauline | BAYART |
| Brice | BAZIN |
| Arash | BEBAHANI |
| Jean-Pierre | BECQWORT |
| Houssem | BEN ALI |
| Emmanuel | BEN SOUSSAN |
| Coralie | BENARD |
| Vincent | BENET |
| Corinne | BENGUIGUI |
| Abdeslam | BENTAL |
| Sara | BENTALEB-BELLATI |
| Isabelle | BERKELMANS |
| Jacques | BERNET |
| Karine | BERNOU |
| Nathalie | BERTIAUX-VANDAELE |
| Pauline | BERTOT |
| Valérie | BERTRAND |
| Emilie | BILOUD |
| Nathalie | BIRON |
| Benjamin | BISMUTH |
| Cyril | BLANCHARD |
| Maurice | BLEUEZ |
| Fabienne | BLONDEL |
| Valérie | BLONDIN |
| Marius | BOBULA |
| Philippe | BOHONT |
| Eléonore | BOIVIN |
| Vanessa | BON DJEMAH |
| Eric | BONIFACE |
| Philippe | BONNIERE |
| Pierre | BONVARLET |
| Arnaud | BORUCHOWICZ |
| Raoul | BOSTVIRONNOIS |
| Médina | BOUALIT |
| Ahlem | BOUAZZA |
| Bruno | BOUCHE |
| Christian | BOUDAILLER |
| Claude | BOURGEAUX |
| Morgane | BOURGEOIS-FUMERY |
| Arnaud | BOURGUET |
| Agnès | BOURIENNE |
| Hamza | BOUTALEB |
| Alexis | BOUTHORS |
| Julien | BRANCHE |
| Franck | BRAZIER |
| Marie | BRIDENNE |
| Hélène | BRIHIER |
| Laura | BRIL |
| Philippe | BULOIS |
| Pierre | BURGIERE |
| Joël | BUTEL |
| Jean-Yves | CANVA |
| Valérie | CANVA-DELCAMBRE |
| Florence | CARDOT |
| Sandrine | CARETTE |
| Pierre | CARPENTIER |
| Michel | CASSAGNOU |
| Jean-François | CASSAR |
| François | CASTEX |
| Pascale | CATALA |
| Stéphane | CATTAN |
| Sylviane | CATTEAU |
| Bernard | CAUJOLLE |
| Gérard | CAYRON |
| Catherine | CHANDELIER |
| Cloé | CHARPENTIER |
| Marthe | CHAVANCE-THELU |
| Agathe | CHENY |
| Dinu | CHIRITA |
| Antoine | CHOTEAU |
| Jean-François | CLAERBOUT |
| Pierre-Yves | CLERGUE |
| Hugues | COEVOET |
| Gil | COHEN |
| Marie | COLIN |
| Régis | COLLET |
| Jean-Frédéric | COLOMBEL |
| Stéphanie | COOPMAN |
| Lucie | CORDIEZ |
| Antoine | CORTOT |
| Jean | CORVISART |
| Frédéric | COUTTENIER |
| Jean-François | CRINQUETTE |
| Valérie | CROMBE |
| Abdelhakim | DAOUDI |
| Vincent | DAPVRIL |
| Thierry | DAVION |
| Sébastien | DECOSTER |
| Laurent | DEFONTAINE |
| Nicolas | DEGRAVE |
| Aurélien | DEJAEGER |
| Richard | DELCENSERIE |
| Marine | DELEPLANQUE |
| Dorothée | DELESALLE |
| Olivier | DELETTE |
| Thierry | DELGRANGE |
| Laurence | DELHOUSTAL |
| Jean-Stéphane | DELMOTTE |
| Sabri | DEMMANE |
| Guy | DEREGNAUCOURT |
| Constance | DESCHEPPER |
| Jean-Pierre | DESECHALLIERS |
| Patrick | DESMET |
| Pierre | DESREUMAUX |
| Gérard | DESSEAUX |
| Philippe | DESURMONT |
| Alain | DEVIENNE |
| Eve | DEVOUGE |
| Alex | DEVROUX |
| Arnaud | DEWAILLY |
| Sébastien | DHARANCY |
| Aude | DI FIORE |
| Emmanuel | DIAZ |
| Djamal-Dine | DJEDDI |
| Rachid | DJEDIR |
| Wissam | DOLEH |
| Marie-Laure | DREHER-DUWAT |
| Richard | DUBOIS |
| Clothilde | DUBURQUE |
| Frédéric | DUCROT |
| Philippe | DUCROTTE |
| André | DUFILHO |
| Christian | DUHAMEL |
| Caroline | DUMANT-FOREST |
| Jean-Louis | DUPAS |
| Frédéric | DUPONT |
| Yves | DURANTON |
| Arnaud | DURIEZ |
| Nicolas | DUVEAU |
| Mohammadi | EL FARISI |
| Khalil | EL HACHKAR |
| Caroline | ELIE |
| Marie-Claire | ELIE-LEGRAND |
| Matthieu | EOCHE |
| Essmaeel | ESSMAEEL |
| Dominique | EVRARD |
| Jean-Paul | EVRARD |
| Armelle | FATOME |
| Karima | FELLAH-SEKKAI |
| Bernard | FILOCHE |
| Laurent | FINET |
| Mathilde | FLAHAUT |
| Camille | FLAMME |
| David | FOISSEY |
| Peggy | FOURNIER |
| Philippe | FOUTREIN |
| Marie-Christine | FOUTREIN-COMES |
| Thierry | FRERE |
| Mathurin | FUMERY |
| Julie | GALAND |
| Philippe | GALLAIS |
| Claudine | GAMBLIN |
| Serge | GANGA |
| Romain | GERARD |
| Guillaume | GESLIN |
| Yves | GHEYSSENS |
| Salah | GHRIB |
| Thierry | GILBERT |
| Bénédicte | GILLET |
| Denis | GODART |
| Jean-Michel | GODCHAUX |
| Guetty | GOEGUEBEUR |
| Odile | GORIA |
| Frédéric | GOTTRAND |
| Philippe | GOWER |
| Lucien | GRADOS |
| Brigitte | GRANDMAISON |
| Marion | GROUX |
| Claire | GUEDON |
| Loïc | GUERBEAU |
| Mathilde | GUEROULT-DERO |
| Jean-François | GUILLARD |
| Laurence | GUILLEM |
| François | GUILLEMOT |
| Dominique | GUIMBER |
| Baya | HADDOUCHE |
| Vincent | HAUTEFEUILLE |
| Philippe | HECKETSWEILLER |
| Geneviève | HECQUET |
| Jean-Pierre | HEDDE |
| Hassina | HELLAL |
| Pierre-Emmanuel | HENNERESSE |
| Michel | HERAUD |
| Sophie | HERVE |
| Bruno | HEYMAN |
| Patrick | HOCHAIN |
| Philippe | HOUCKE |
| Lucie | HOUSSIN-BAILLLY |
| Bruno | HUGUENIN |
| Silviu | IOBAGIU |
| Shata | ISTANBOLI |
| Alexsandar | IVANOVIC |
| Isabelle | IWANICKI-CARON |
| Eric | JANICKI |
| Marine | JARRY |
| Charlotte | JEAN BART |
| Claude | JONAS |
| Julia | JOUGON |
| Anne | JOUVENET |
| Naeim | KASSAR |
| Fadi | KATHERIN |
| Alfred | KERLEVEO |
| Ali | KHACHFE |
| Alfred | KIRIAKOS |
| Jean | KIRIAKOS |
| Olivier | KLEIN |
| Matthieu | KOHUT |
| Richard | KORNHAUSER |
| Demetrios | KOUTSOMANIS |
| Jean-Eric | LABERENNE |
| Eric | LACOTTE |
| Guy | LAFFINEUR |
| Marine | LAGARDE |
| Anouck | LAHAYE |
| Arnaud | LALANNE |
| Ambroise | LALIEU |
| Pierre | LANNOY |
| José | LAPCHIN |
| Michel | LAPRAND |
| Denis | LAUDE |
| Christian | LE COUTEULX |
| Charles | LE GOFFIC |
| Alain | LE GRIX |
| Jean-Philippe | LE MOUEL |
| Pauline | LE ROY |
| Rachida | LEBLANC |
| Paul | LECIEUX |
| Stéphane | LECLEIRE |
| Nathalie | LECLERC |
| Jean | LEDENT |
| Jean | LEFEBVRE |
| Pascale | LEFILLIATRE |
| Céline | LEGRAND |
| Patrick | LELONG |
| Bernard | LELUYER |
| Caroline | LEMAITRE |
| Lucie | LEPILEUR |
| Antoine | LEPLAT |
| Elodie | LEPOUTRE-DUJARDIN |
| Gabriel | LEPPEUT |
| Henri | LEROI |
| Maryvonne | LEROY |
| Benoît | LESAGE |
| Jocelyn | LESAGE |
| Xavier | LESAGE |
| Isabelle | LESCANNE-DARCHIS |
| Dominique | LESCUT |
| Bruno | LEURENT |
| Delphine | LEY |
| Michel | LHERMIE |
| Louise | LIBIER |
| Bernard | LISAMBERT |
| Isabelle | LOGE |
| Julien | LOREAU |
| Alexandre | LOUVET |
| Joséphine | LOZINGUEZ |
| Henri | LUBREZ |
| Damien | LUCIDARME |
| Jean-Jacques | LUGAND |
| Olivier | MACAIGNE |
| Denis | MAETZ |
| Dominique | MAILLARD |
| Hubert | MANCHERON |
| Olivia | MANOLACHE |
| Anne-Bérengère | MARKS-BRUNEL |
| Charline | MARRE |
| Raymond | MARTI |
| Eric | MARZLOFF |
| Philippe | MATHURIN |
| Jacques | MAUILLON |
| Vincent | MAUNOURY |
| Jean-Luc | MAUPAS |
| Michèle-Ange | MEDAM DJOMO |
| Chloé | MELCHIOR |
| Ziad | MELKI |
| B | MESNARD |
| Patrice | METAYER |
| Lofti | METHARI |
| Franck | MEURISSE |
| Laurent | MICHAUD |
| Patricia | MODAINE |
| Angélique | MONTHE |
| Loïk | MOREL |
| Mathilde | MORIN |
| Pierre-Eugène | MORTIER |
| Perrine | MORTREUX |
| Olivier | MOUTERDE |
| Nicolas | MOZZICONACI |
| Jean | MUDRY |
| Maria | NACHURI |
| Minh Dung | NGO |
| Eric | N'GUYEN KHAC |
| Bertrand | NOTTEGHEM |
| Vincent | OLLEVIER |
| Atika | OURAGHI |
| Barriza | OUSSADOU |
| Dominique | OUVRY |
| Bernard | PAILLOT |
| Claire | PAINCHART |
| Nicole | PANIEN-CLAUDOT |
| Christian | PAOLETTI |
| Arsène | PAPAZIAN |
| Bruno | PARENT |
| Jean-Claude | PARIS |
| Philippe | PATRIER |
| Thierry | PAUPARD |
| Bernard | PAUWELS |
| Mathieu | PAUWELS |
| Richard | PETIT |
| Muriel | PIAT |
| Sandrine | PIOTTE |
| Christophe | PLANE |
| Bernard | PLOUVIER |
| Eric | POLLET |
| Pierre | POMMELET |
| Daniela | POP |
| Charlotte | PORDES |
| Gérard | POUCHAIN |
| Philippe | PRADES |
| Jean-Christophe | PREVOST |
| Manon | PRUIT |
| Gilles | QUARTIER |
| Anne-Marie | QUEUNIET |
| Jean-François | QUINTON |
| Alain | RABACHE |
| Gilles | RACLOT |
| Sébastien | RATAJCZYK |
| Nicole | REIX |
| Thibaud | RENAUT-VANTROYS |
| Marine | REVILLION |
| Ghassan | RIACHI |
| Clémentine | RIAULT |
| Nicolas | RICHARD |
| Cécile | RICHEZ |
| Benoît | RIMBERT |
| Philippe | ROBINSON |
| Juan Daniel | RODRIGUEZ |
| Jean | ROGER |
| Jean-Marc | ROUX |
| Alain | RUDELLI |
| Clémence | SAINGIER |
| Guillaume | SAVOYE |
| Patrick | SCHLOSSBERG |
| David | SEFRIOUI |
| Michel | SEGRESTIN |
| David | SEGUY |
| Célik | SEMINUR |
| François | SEVENET |
| Jean | SILVIE |
| Claire | SPYCKERELLE |
| Nathalie | TALBODEC |
| Noémie | TAVERNIER |
| Henriette | TCHANDEU |
| Aurore | TECHY |
| Jean-Luc | THELU |
| Henri | THIEBAULT |
| Jean-Marie | THOREL |
| Christophe | THUILLIER |
| Guillaume | TIELMAN |
| Manuella | TODE |
| Jean | TONNEL |
| Jean-Yves | TOUCHAIS |
| Audrey | TOULEMONDE-HUGUET |
| Pierre | TOUMELIN |
| Yvan | TOUZE |
| Léa | TRAN |
| Jean-Luc | TRANVOUEZ |
| Nadia | TRIKI |
| Dominique | TURCK |
| Justine | TURPIN |
| Eric | VAILLANT |
| Claude | VALMAGE |
| Dominique | VANCO |
| Nathalie | VANDAELE-BERTIAUX |
| Hélène | VANDAMME |
| Elise | VANDER EEKEN |
| Etienne | VANDERBERCQ |
| Philippe | VANDERMOLLEN |
| Philippe | VANDEVENNE |
| Lionel | VANDEVILLE |
| Alain | VANDEWALLE |
| Jean-Pierre | VANHOOVE |
| Audrey | VANRENTERGHEM |
| Charlotte | VANVEUREN |
| Iona | VASIES |
| Guy | VERBIESE |
| Juliette | VERLYNDE |
| Philippe | VERMELLE |
| Christine | VERNE |
| Gwenola | VERNIER-MASSOUILLE |
| Perrine | VEZELIER-COCQ |
| Juliette | VIART |
| Benoît | VIGNERON |
| Marc | VINCENDET |
| Jacques | VIOT |
| YM | VOIMENT |
| Jean-Yves | WALLEZ |
| Michel | WANTIER |
| Faustine | WARTEL |
| Jean-Christian | WEBER |
| Jean-Louis | WILLOCQUET |
| Nathalie | WIZLA |
| Eric | WOLSCHIES |
| Tajiogue | YIMFOR |
| Oana | ZAHARA |
| Alberto | ZALAR |
| Sonia | ZAOUI |
| Anne | ZELLWEGER |
