# Supplementary material for: Incidence, prevalence and clinical presentation of inflammatory bowel diseases in Northern France: a 30-year population-based study
Source: Lancet Reg Health Eur. 2024 Oct 18;47:101097. doi: 10.1016/j.lanepe.2024.101097 (PMC11522416; doi:10.1016/j.lanepe.2024.101097)
Supplement: Supplementary Figs. S1 and S2 and Tables S1–S7 [file mmc2.docx]

**Supplementary material**

**Supplementary Table 1**: Incidence rates /10^5^ person-years over the 1988-2017 study period and incidence rate ratio (IRR) according to gender and age group in CD and UC.

|  |  | **1988-2017 Incidence rate /10^5^ person-years [95% CI]** | **Incidence Rate Ratio (IRR) [95% CI]** | **p-value** |
| --- | --- | --- | --- | --- |
|  | **Male** | 6·4 [6·3 ; 6·6] | Ref |  |
|  | **Female** | 8·0 [7·8 ; 8·2] | 1·25 [1·19 ; 1·31] | <0·0001 |
| **CD** | **<17 years** | 3·7 [3·5 ; 3·9] | Ref |  |
|  | **17-39 years** | 15·4 [15·1 ; 15·8] | 4·28 [3·94 ; 4·65] | <0·0001 |
|  | **40-59 years** | 5·3 [5·1 ; 5·6] | 1·43 [1·29 ; 1·58] | <0·0001 |
|  | **60 and over** | 2·4 [2·3 ; 2·6] | 0·63 [0·56 ; 0·72] | <0·0001 |
|  | **Male** | 5·7 [5·5 ; 5·9] | Ref |  |
|  | **Female** | 4·5 [4·4 ; 4·7] | 0·83 [0·78 ; 0·89] | <0·0001 |
| **UC** | **<17 years** | 1·4 [1·3 ; 1·5] | Ref |  |
|  | **17-39 years** | 8·7 [8·4 ; 8·9] | 6·26 [5·41 ; 7·25] | <0·0001 |
|  | **40-59 years** | 5·6 [5·4 ; 5·8] | 4·07 [3·49 ; 4·74] | <0·0001 |
|  | **60 and over** | 3·1 [2·9 ; 3·3] | 2·28 [1·92 ; 2·71] | <0·0001 |

**Supplementary Table 2**: Sociodemographic and clinical data for patients with IBD from a population-based registry in northern France from 1988 to 2017, by age group (n=22,879).

| **Variables at diagnosis** | |  |  | **<17**  **(n=2,103)** | **17-39 (n=13,894)** | **40-59 (n=4,905)** | **≥ 60 (n=1,977)** | **P-value** |
| --- | --- | --- | --- | --- | --- | --- | --- | --- |
| **Type of IBD** |  |  |  |  |  |  |  |  |
| CD |  |  |  | 1,510 (71·8%) | 8,796 (63·3%) | 2,318 (47·3%) | 821 (41·5%) | <0·0001 |
| UC |  |  |  | 562 (26·7%) | 4,766 (34·3%) | 2,424 (49·4%) | 1,051 (53·2%) |  |
| IBDU |  |  |  | 31 (1·5%) | 332 (2·4%) | 163 (3·3%) | 105 (5·3%) |  |
| **CD** |  |  |  |  |  |  |  |  |
| Family history of IBD | |  |  | 276 (18·3%) | 1,168 (13·3%) | 212 (9·1%) | 44 (5·4%) | <0·0001 |
| Women |  |  |  | 672 (44·5%) | 5,150 (58·6%) | 1,229 (53·0%) | 484 (58·9%) | <0·0001 |
| Median [IQR] time between symptoms onset and diagnosis, months | | | | 3 [2 ; 7] | 3 [1; 9] | 3 [1; 9] | 3 [1; 8] | 0·101 |
| Disease site* |  |  |  |  |  |  |  |  |
| L1 |  |  |  | 222 (15·5%) | 1,785 (20·9%) | 531 (23·6%) | 125 (15·8%) | <0·0001 |
| L2 |  |  |  | 378 (26·0%) | 2,213 (25·9%) | 858 (38·0%) | 464 (58·5%) |  |
| L3 |  |  |  | 849 (58·5%) | 4,549 (53·2%) | 865 (38·4%) | 204 (25·7%) |  |
| L4 |  |  |  | 482 (31·9%) | 2,208 (23·1%) | 343 (14·8%) | 98 (11·9%) | <0·0001 |
| Behavior* ^£^ |  |  |  |  |  |  |  |  |
| B1 |  |  |  | 665 (82·8%) | 2386 (73·1%) | 594 (70·2%) | 195 (67·5%) | <0·0001 |
| B2 |  |  |  | 105 (13·1%) | 584 (17·9%) | 175 (20·7%) | 64 (22·1%) |  |
| B3 |  |  |  | 33 (4·1%) | 293 (9·0%) | 77 (9·1%) | 30 (10·4%) |  |
| Perianal disease | |  |  | 92 (6·1%) | 398 (4·5%) | 118 (5·1%) | 50 (6·1%) | 0·019 |
| Extra-intestinal manifestations |  |  |  | 340 (22·5%) | 924 (10·5%) | 246 (10·6%) | 55 (6·7%) | <0·0001 |
| **UC** |  |  |  |  |  |  |  |  |
| Family history of IBD | |  |  | 80 (14·2%) | 372 (7·8%) | 109 (4·5%) | 33 (3·1%) | <0·0001 |
| Women |  |  |  | 317 (56·4%) | 2,472 (51·9%) | 890 (36·7%) | 425 (40·4%) | <0·0001 |
| Median [IQR] time between symptoms onset and diagnosis, months | | | | 2 [1 ; 5] | 2 [1; 6] | 2 [1; 6] | 2 [1; 5] | 0·636 |
| Disease site* |  |  |  |  |  |  |  |  |
| E1 |  |  |  | 122 (22·1%) | 1,895 (40·2%) | 925 (38·7%) | 215 (20·6%) | <0·0001 |
| E2 |  |  |  | 171 (30·9%) | 1,537 (32·7%) | 934 (39·0%) | 568 (54·6%) |  |
| E3 |  |  |  | 260 (47·0%) | 1,278 (27·1%) | 533 (22·3%) | 258 (24·8%) |  |
| Extra-intestinal manifestations |  |  |  | 34 (6·0%) | 162 (3·4%) | 71 (2·9%) | 32 (3·0%) | 0·003 |

** According to the Montreal classification*

*^£^ Recorded in the EPIMAD registry since 2008*

**Supplementary Table 3**: Time trends in incidence rates of IBD from a prospective population-based registry in northern France from 1988 to 2017 by sex and by age group (n=22,879).

| **Sex** | **Age group** | **Standardized incidence/10^5^** | | **APC   % / year** | **P-value** |
| --- | --- | --- | --- | --- | --- |
|  |  | **1988-1990** | **2015-2017** |  |  |
| **Both** | <17 years | 3·1 [2·6 ; 3·7] | 8·6 [7·7 ; 9·5] | **+4·6 [3·9 ; 5·2]***** | **<·0001** |
|  | 17-39 years | 18·8 [17·7 ; 20·1] | 27·5 [26·1 ; 28·9] | **+1·6 [1·4 ; 1·8]***** | **<·0001** |
|  | 40-59 years | 10·0 [8·9 ; 11·2] | 11·4 [10·4 ; 12·4] | **+0·5 [0·1 ; 0·9]*** | **0·012** |
|  | ≥60 years | 6·5 [5·5 ; 7·6] | 6·0 [5·3 ; 6·8] | -0·5 [-1·1 ; 0·1] | 0·131 |
|  | All | 10·4 [9·9 ; 10·9] | 14·1 [13·6 ; 14·7] | **+1·5 [1·2; 1·8] ***** | <0·0001 |
|  | <17 years | \| 3·2 [2·4 ; 4·1] \| \| --- \| | 8·1 [6·8 ; 9·5] | **+4·3 [3·4 ; 5·2]***** | **<·0001** |
| **Women** | 17-39 years | 20·7 [19·0 ; 22·5] | 29·5 [27·5 ; 31·7] | **+1·8 [1·5 ; 2·1]***** | **<·0001** |
|  | 40-59 years | 7·8 [6·4 ; 9·3] | 11·2 [9·9 ; 12·6] | **+1·4 [0·8 ; 2]***** | **<·0001** |
|  | ≥60 years | 5·6 [4·4 ; 7·0] | 5 [4·1 ; 6·1] | -0·5 [-1·3 ; 0·4] | 0·281 |
|  | All | 10·1 [9·4 ; 10·9] | 14·3 [13·6 ; 15·1] | **+1·6 [1·2 ; 2·1]***** | **<·0001** |
|  | <17 years | 3·0 [2·3 ; 3·8] | 9·1 [7·8 ; 10·5] | **+4·8 [3·9 ; 5·7]***** | **<·0001** |
| **Men** | 17-39 years | 17·0 [15·5 ; 18·7] | 25·5 [23·6 ; 27·5] | **+1·4 [1 ; 1·7]***** | **<·0001** |
|  | 40-59 years | 12·3 [10·6 ; 14·2] | 11·5 [10·2 ; 13] | -0·1 [-0·7 ; 0·4] | 0·609 |
|  | ≥60 years | 8·0 [6·3 ; 10·2] | 7·1 [5·9 ; 8·4] | -0·6 [-1·4 ; 0·3] | 0·181 |
|  | All | 10·9 [10·0 ; 11·8] | 14·0 [13·2 ; 14·8] | **+1·3 [0·8 ; 1·7]***** | **<·0001** |

* significant time trend, 0·001≤p<0·05

** significant time trend, 0·0001≤p<0·001

*** significant time trend, p<0·0001

APC: annual percent change estimated using a log-linear Poisson model

**Supplementary Table 4**: Time trends in incidence rates of CD from a prospective population-based registry in northern France from 1988 to 2017 by sex and by age group (n=13,445).

| **Sex** | **Age group** | **Standardized incidence/10^5^** | | **APC   % / year** | **P-value** |
| --- | --- | --- | --- | --- | --- |
|  |  | **1988-1990** | **2015-2017** |  |  |
|  | <17 years | 2·2 [1·7 ; 2·7] | 5·7 [5·0 ; 6·5] | **+4·3 [3·5 ; 5·1]***** | **<·0001** |
|  | 17-39 years | 10·6 [9·7 ; 11·5] | 16·7 [15·6 ; 17·8] | **+1·9 [1·5 ; 2·2]***** | **<·0001** |
|  | 40-59 years | 3·8 [3·2 ; 4·6] | 5·1 [4·5 ; 5·8] | **+0·9 [0·3 ; 1·5]**** | **0·006** |
|  | ≥60 years | 2·6 [2·0 ; 3·4] | 2·4 [2·0 ; 3·0] | +0·1 [-1·0 ; 1·1] | 0·893 |
| **Both** | All | 5·1 [4·8 ; 5·5] | 7·9 [7·4 ; 8·3] | **+1·9 [1·6 ; 2·2]***** | **<·0001** |
|  | <17 years | 2·1 [1·5 ; 2·9] | 4·9 [4·0 ; 6·0] | **+3·9 [2·8 ; 5·1]***** | **<·0001** |
| **Women** | 17-39 years | 12·9 [11·5 ; 14·3] | 18·2 [16·6 ; 20·0] | **+1·8 [1·4 ; 2·2]***** | **<·0001** |
|  | 40-59 years | 4·1 [3·1 ; 5·2] | 5·8 [4·9 ; 6·9] | **+1·3 [0·5 ; 2·2]*** | **0·002** |
|  | ≥60 years | 2·8 [2·0 ; 3·9] | 2·3 [1·7 ; 3·0] | -0·5 [-1·7 ; 0·8] | 0·477 |
|  | All | 5·9 [5·4 ; 6·5] | 8·3 [7·7 ; 8·9] | **+1·7 [1·3 ; 2·1]***** | **<·0001** |
|  | <17 years | 2·2 [1·6 ; 3·0] | 6·5 [5·5 ; 7·8] | **+4·6 [3·6 ; 5·5]***** | **<·0001** |
| **Men** | 17-39 years | 8·3 [7·2 ; 9·4] | 15·1 [13·6 ; 16·7] | **+2·0 [1·5 ; 2·4]***** | **<·0001** |
|  | 40-59 years | 3·6 [2·7 ; 4·7] | 4·4 [3·5 ; 5·3] | +0·4 [-0·4 ; 1·2] | 0·347 |
|  | ≥60 years | 2·4 [1·4 ; 3·9] | 2·5 [1·9 ; 3·4] | +0·8 [-0·6 ; 2·3] | 0·244 |
|  | All | 4·4 [3·9 ; 5·0] | 7·4 [6·8 ; 8·0] | **+2·0 [1·6 ; 2·5]***** | **<·0001** |

* significant time trend, 0·001≤p<0·05

** significant time trend, 0·0001≤p<0·001

*** significant time trend, p<0·0001

APC: annual percent change estimated using a log-linear Poisson model

**Supplementary Table 5**: Time trends in incidence rates of UC from a prospective population-based registry in northern France from 1988 to 2017 by sex and by age group (n=8,803).

| **Sex** | **Age group** | **Standardized incidence/10^5^** | | **APC   % / year** | **P-value** |
| --- | --- | --- | --- | --- | --- |
|  |  | **1988-1990** | **2015-2017** |  |  |
| **Both** | <17 years | 0·8 [0·6 ; 1·2] | 2·7 [2·2 ; 3·3] | **+5·4 [4·3 ; 6·6]***** | **<·0001** |
|  | 17-39 years | 7·4 [6·7 ; 8·2] | 10·6 [9·7 ; 11·5] | **+1·5 [1·2 ; 1·9]***** | **<·0001** |
|  | 40-59 years | 4·9 [4·2 ; 5·8] | 6·1 [5·4 ; 6·8] | +0·7 [0·1 ; 1·2]* | 0·014 |
|  | ≥60 years | 3·3 [2·6 ; 4·1] | 3·4 [2·9 ; 4·1] | -0·2 [-1 ; 0·5] | 0·53 |
|  | All | 4·5 [4·1 ; 4·9] | 6·1 [5·7 ; 6·5] | **+1·3 [0·9 ; 1·7]***** | **<·0001** |
|  | <17 years | 1·0 [0·6 ; 1·5] | 3·0 [2·3 ; 3·9] | **+5·2 [3·9 ; 6·6]***** | **<·0001** |
| **Women** | 17-39 years | 6·9 [5·9 ; 8·0] | 11·0 [9·8 ; 12·4] | **+2·1 [1·7 ; 2·6]***** | **<·0001** |
|  | 40-59 years | 3·1 [2·3 ; 4·1] | 5·2 [4·3 ; 6·2] | **+1·7 [1 ; 2·5]***** | **<·0001** |
|  | ≥60 years | 2·1 [1·5 ; 3·1] | 2·6 [2·0 ; 3·4] | +0·4 [-0·7 ; 1·5] | 0·504 |
|  | All | 3·6 [3·1 ; 4·0] | 5·8 [5·3 ; 6·3] | **+1·9 [1·3 ; 2·6]***** | **<·0001** |
|  | <17 years | 0·7 [0·4 ; 1·2] | 2·4 [1·8 ; 3·2] | **+5·8 [4 ; 7·5]***** | **<·0001** |
| **Men** | 17-39 years | 7·9 [6·8 ; 9·1] | 10·2 [9 ; 11·5] | **+0·9 [0·4 ; 1·4]*** | **0·001** |
|  | 40-59 years | 6·9 [5·6 ; 8·3] | 7·0 [6·0 ; 8·2] | +0·1 [-0·6 ; 0·7] | 0·883 |
|  | ≥60 years | 4·9 [3·7 ; 6·7] | 4·4 [3·5 ; 5·5] | -0·8 [-1·7 ; 0·2] | 0·118 |
|  | All | 5·5 [5·0 ; 6·2] | 6·4 [5·9 ; 7·0] | **+0·8 [0·2 ; 1·3]*** | **0·006** |

* significant time trend, 0·001≤p<0·05

** significant time trend, 0·0001≤p<0·001

*** significant time trend, p<0·0001

APC: annual percent change estimated using a log-linear Poisson model

**Supplementary Table 6**: Incidences rates of CD (n=13,445) and UC (n=8,803) with 95% CI in Northern France over the study period (1988-2017), by sex and 5-year age groups.

| **Sex** | **Age-group** | **Crohn** | | | **Ulcerative Colitis** | | |
| --- | --- | --- | --- | --- | --- | --- | --- |
|  |  | **Incidence rate** | **Lower bound of CI** | **Upper bound of CI** | **Incidence rate** | **Lower bound of CI** | **Upper bound of CI** |
| Women | 0-4 | **0·14** | 0·04 | 0·24 | **0·18** | 0·07 | 0·29 |
| Women | 5-9 | **1·35** | 1·06 | 1·65 | **0·91** | 0·66 | 1·15 |
| Women | 10-14 | **4·54** | 4·00 | 5·08 | **2·33** | 1·94 | 2·72 |
| Women | 15-19 | **20·22** | 19·10 | 21·34 | **5·55** | 4·97 | 6·14 |
| Women | 20-24 | **28·72** | 27·37 | 30·06 | **9·60** | 8·83 | 10·38 |
| Women | 25-29 | **18·54** | 17·44 | 19·64 | **11·37** | 10·51 | 12·23 |
| Women | 30-34 | **13·28** | 12·35 | 14·21 | **9·28** | 8·50 | 10·05 |
| Women | 35-39 | **9·70** | 8·91 | 10·49 | **7·31** | 6·63 | 8·00 |
| Women | 40-44 | **8·29** | 7·57 | 9·02 | **5·47** | 4·88 | 6·07 |
| Women | 45-49 | **5·98** | 5·34 | 6·62 | **4·32** | 3·78 | 4·87 |
| Women | 50-54 | **4·33** | 3·76 | 4·89 | **3·23** | 2·74 | 3·72 |
| Women | 55-59 | **3·54** | 3·01 | 4·07 | **3·13** | 2·63 | 3·63 |
| Women | 60-64 | **2·76** | 2·27 | 3·25 | **2·85** | 2·36 | 3·34 |
| Women | 65-69 | **2·52** | 2·04 | 3·01 | **2·48** | 1·99 | 2·96 |
| Women | 70-74 | **2·91** | 2·35 | 3·48 | **2·31** | 1·80 | 2·81 |
| Women | 75-79 | **2·56** | 1·99 | 3·13 | **1·77** | 1·30 | 2·25 |
| Women | 80-84 | **2·17** | 1·58 | 2·76 | **1·71** | 1·19 | 2·23 |
| Women | ≥85 | **1·17** | 0·72 | 1·62 | **0·94** | 0·54 | 1·35 |
| Men | 0-4 | **0·20** | 0·09 | 0·32 | **0·10** | 0·02 | 0·18 |
| Men | 5-9 | **1·39** | 1·09 | 1·68 | **0·38** | 0·22 | 0·53 |
| Men | 10-14 | **6·76** | 6·11 | 7·40 | **1·69** | 1·37 | 2·02 |
| Men | 15-19 | **15·19** | 14·24 | 16·15 | **5·06** | 4·51 | 5·61 |
| Men | 20-24 | **18·18** | 17·12 | 19·24 | **7·77** | 7·08 | 8·47 |
| Men | 25-29 | **14·09** | 13·12 | 15·05 | **9·16** | 8·39 | 9·94 |
| Men | 30-34 | **10·35** | 9·53 | 11·17 | **9·48** | 8·69 | 10·27 |
| Men | 35-39 | **7·37** | 6·68 | 8·07 | **8·57** | 7·83 | 9·31 |
| Men | 40-44 | **5·96** | 5·34 | 6·58 | **8·08** | 7·35 | 8·80 |
| Men | 45-49 | **5·22** | 4·61 | 5·82 | **7·62** | 6·90 | 8·35 |
| Men | 50-54 | **4·80** | 4·19 | 5·40 | **6·42** | 5·72 | 7·12 |
| Men | 55-59 | **4·51** | 3·89 | 5·12 | **6·79** | 6·04 | 7·55 |
| Men | 60-64 | **3·00** | 2·46 | 3·54 | **5·24** | 4·53 | 5·95 |
| Men | 65-69 | **3·01** | 2·42 | 3·60 | **6·11** | 5·27 | 6·96 |
| Men | 70-74 | **1·96** | 1·41 | 2·51 | **4·33** | 3·51 | 5·14 |
| Men | 75-79 | **2·35** | 1·65 | 3·04 | **3·84** | 2·95 | 4·73 |
| Men | 80-84 | **1·48** | 0·79 | 2·16 | **2·38** | 1·51 | 3·25 |
| Men | ≥85 | **0·87** | 0·23 | 1·51 | **0·75** | 0·15 | 1·34 |

**Supplementary Table 7**: Standardized incidence rates with confidence intervals for CD (n=13,445) and UC n=8,803) in Northern France from 1988 to 2017 by sex and age group.

| **Group** | **Period** | **Crohn** | | | **Ulcerative Colitis** | | |
| --- | --- | --- | --- | --- | --- | --- | --- |
|  |  | **Standardized incidence rate** | **Lower bound of CI** | **Upper bound of CI** | **Standardized incidence rate** | **Lower bound of CI** | **Upper bound of CI** |
| Women | 1988-1990 | **5·9** | 5·4 | 6·5 | **3·6** | 3·1 | 4·0 |
| Women | 1991-1993 | **5·8** | 5·3 | 6·3 | **3·8** | 3·4 | 4·2 |
| Women | 1994-1996 | **6·6** | 6·1 | 7·2 | **3·7** | 3·3 | 4·1 |
| Women | 1997-1999 | **7·7** | 7·2 | 8·3 | **3·8** | 3·4 | 4·3 |
| Women | 2000-2002 | **8·1** | 7·5 | 8·7 | **4·2** | 3·7 | 4·6 |
| Women | 2003-2005 | **8·5** | 7·9 | 9·1 | **4·9** | 4·4 | 5·4 |
| Women | 2006-2008 | **9·9** | 9·2 | 10·5 | **5·0** | 4·6 | 5·5 |
| Women | 2009-2011 | **9·2** | 8·6 | 9·9 | **5·3** | 4·8 | 5·8 |
| Women | 2012-2014 | **9·8** | 9·2 | 10·5 | **5·4** | 5·0 | 6·0 |
| Women | 2015-2017 | **8·3** | 7·7 | 8·9 | **5·8** | 5·3 | 6·3 |
| Men | 1988-1990 | **4·4** | 3·9 | 5·0 | **5·5** | 5·0 | 6·2 |
| Men | 1991-1993 | **5·4** | 4·9 | 5·9 | **5·9** | 5·3 | 6·5 |
| Men | 1994-1996 | **5·4** | 4·9 | 5·9 | **5·3** | 4·8 | 5·8 |
| Men | 1997-1999 | **6·4** | 5·8 | 6·9 | **5·2** | 4·7 | 5·8 |
| Men | 2000-2002 | **6·4** | 5·9 | 6·9 | **5·0** | 4·5 | 5·6 |
| Men | 2003-2005 | **6·7** | 6·2 | 7·3 | **6·1** | 5·5 | 6·6 |
| Men | 2006-2008 | **7·3** | 6·7 | 7·9 | **6·3** | 5·8 | 6·9 |
| Men | 2009-2011 | **7·4** | 6·8 | 8·0 | **6·0** | 5·4 | 6·5 |
| Men | 2012-2014 | **7·7** | 7·2 | 8·3 | **5·5** | 5·0 | 6·1 |
| Men | 2015-2017 | **7·4** | 6·8 | 8·0 | **6·4** | 5·9 | 7·0 |
| 0-16 years | 1988-1990 | **2·2** | 1·7 | 2·7 | **0·8** | 0·6 | 1·2 |
| 0-16 years | 1991-1993 | **2·0** | 1·6 | 2·5 | **0·7** | 0·5 | 1·0 |
| 0-16 years | 1994-1996 | **2·5** | 2·0 | 3·0 | **0·8** | 0·6 | 1·2 |
| 0-16 years | 1997-1999 | **2·9** | 2·4 | 3·5 | **0·9** | 0·7 | 1·3 |
| 0-16 years | 2000-2002 | **2·9** | 2·4 | 3·5 | **0·9** | 0·6 | 1·2 |
| 0-16 years | 2003-2005 | **3·6** | 3·0 | 4·2 | **1·2** | 0·9 | 1·6 |
| 0-16 years | 2006-2008 | **5·0** | 4·3 | 5·7 | **1·7** | 1·3 | 2·1 |
| 0-16 years | 2009-2011 | **5·0** | 4·3 | 5·7 | **2·2** | 1·7 | 2·7 |
| 0-16 years | 2012-2014 | **5·8** | 5·0 | 6·6 | **2·1** | 1·7 | 2·6 |
| 0-16 years | 2015-2017 | **5·7** | 5·0 | 6·5 | **2·7** | 2·2 | 3·3 |
| 17-39 years | 1988-1990 | **10·6** | 9·7 | 11·5 | **7·4** | 6·7 | 8·2 |
| 17-39 years | 1991-1993 | **12·1** | 11·3 | 13·0 | **7·2** | 6·6 | 8·0 |
| 17-39 years | 1994-1996 | **12·9** | 12·0 | 13·8 | **7·8** | 7·1 | 8·5 |
| 17-39 years | 1997-1999 | **15·2** | 14·3 | 16·3 | **7·7** | 7·0 | 8·5 |
| 17-39 years | 2000-2002 | **15·7** | 14·7 | 16·7 | **7·7** | 7·0 | 8·5 |
| 17-39 years | 2003-2005 | **16·1** | 15·1 | 17·2 | **9·6** | 8·8 | 10·5 |
| 17-39 years | 2006-2008 | **19·2** | 18·1 | 20·4 | **10·1** | 9·2 | 11·0 |
| 17-39 years | 2009-2011 | **17·8** | 16·7 | 19·0 | **9·5** | 8·7 | 10·4 |
| 17-39 years | 2012-2014 | **18·7** | 17·6 | 19·9 | **9·4** | 8·6 | 10·3 |
| 17-39 years | 2015-2017 | **16·7** | 15·6 | 17·8 | **10·6** | 9·7 | 11·5 |
| 40-59 years | 1988-1990 | **3·8** | 3·2 | 4·6 | **4·9** | 4·2 | 5·8 |
| 40-59 years | 1991-1993 | **4·4** | 3·8 | 5·1 | **5·7** | 5·0 | 6·6 |
| 40-59 years | 1994-1996 | **4·6** | 4·0 | 5·3 | **5·0** | 4·4 | 5·8 |
| 40-59 years | 1997-1999 | **5·2** | 4·5 | 6·0 | **5·0** | 4·3 | 5·7 |
| 40-59 years | 2000-2002 | **6·0** | 5·3 | 6·8 | **5·0** | 4·4 | 5·7 |
| **Group** | **Period** | **Crohn** | | | **Ulcerative colitis** | | |
|  |  | **Standardized incidence rate** | **Lower bound of CI** | **Upper bound of CI** | **Standardized incidence rate** | **Lower bound of CI** | **Upper bound of CI** |
| 40-59 years | 2003-2005 | **5·9** | 5·3 | 6·7 | **6·2** | 5·5 | 7·0 |
| 40-59 years | 2006-2008 | **5·6** | 4·9 | 6·3 | **6·2** | 5·5 | 7·0 |
| 40-59 years | 2009-2011 | **5·9** | 5·2 | 6·6 | **5·9** | 5·2 | 6·6 |
| 40-59 years | 2012-2014 | **6·2** | 5·5 | 7·0 | **5·9** | 5·2 | 6·6 |
| 40-59 years | 2015-2017 | **5·1** | 4·5 | 5·8 | **6·1** | 5·4 | 6·8 |
| 60 years and more | 1988-1990 | **2·6** | 2·0 | 3·4 | **3·3** | 2·6 | 4·1 |
| 60 years and more | 1991-1993 | **2·1** | 1·6 | 2·7 | **3·7** | 3·0 | 4·4 |
| 60 years and more | 1994-1996 | **2·4** | 1·9 | 3·1 | **2·6** | 2·1 | 3·3 |
| 60 years and more | 1997-1999 | **2·7** | 2·2 | 3·4 | **2·9** | 2·3 | 3·5 |
| 60 years and more | 2000-2002 | **2·1** | 1·6 | 2·6 | **3·1** | 2·6 | 3·8 |
| 60 years and more | 2003-2005 | **2·8** | 2·2 | 3·4 | **2·8** | 2·2 | 3·4 |
| 60 years and more | 2006-2008 | **2·4** | 1·9 | 3·0 | **2·9** | 2·3 | 3·5 |
| 60 years and more | 2009-2011 | **2·5** | 2·0 | 3·1 | **3·3** | 2·8 | 4·0 |
| 60 years and more | 2012-2014 | **2·5** | 2·0 | 3·1 | **2·9** | 2·3 | 3·5 |
| 60 years and more | 2015-2017 | **2·4** | 2·0 | 3·0 | **3·4** | 2·9 | 4·1 |

**Supplementary Figure 1**: Changes over time in the standardized incidence rates for CD (n=13,445) and UC n=8,803) in northern France from 1988 to 2017 by sex and by age group, as recorded in the EPIMAD registry. Each data point corresponds to the mean value for a 3-year period. A) CD incidence rates by age in women. B) CD incidence rates by age in men. C) UC incidence rates by age in women. D) UC incidence rates by age in men.


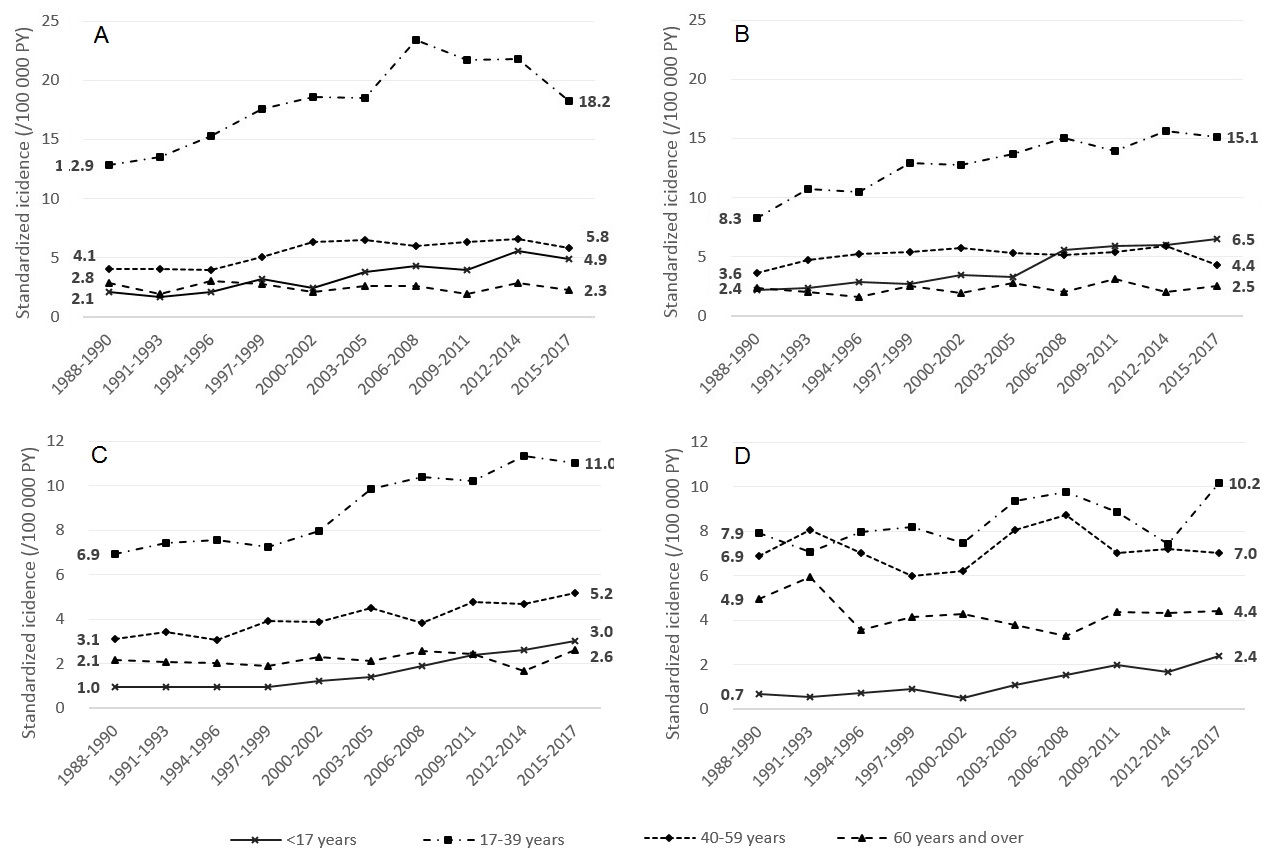


**Supplementary Figure 2**: Changes over time in the incidence rates for pediatric-onset IBD in northern France from 1988 to 2017 by three age group, as recorded in the EPIMAD registry. Each data point corresponds to the mean value for a 3-year period.


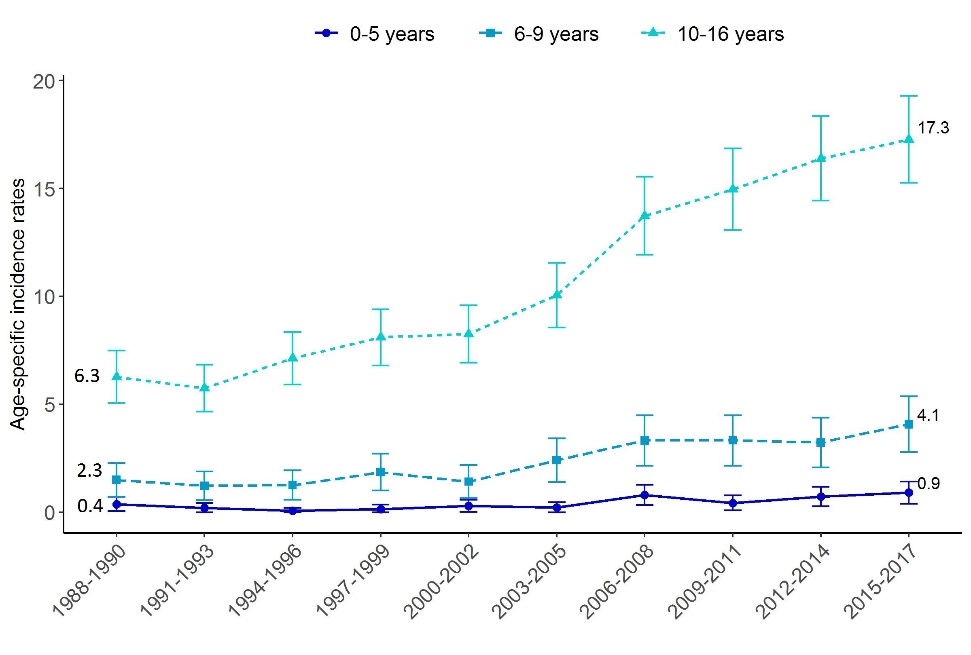


**EPIMAD study group :**

AGOUTE Eric, AL GHOSSAINI Najib, AL HAMEEDI Raied, AL KHATIB Myriam, AL TURK Saria, ANDRE Jean-Marie, ANTOINE Matthieu, ANTONIETTI Michel, AOUAKLI Amar, ARMENGOL-DEBEIR Laura, AROICHANE Ibrahim, ASSI Fadi, AUXENFANTS Eric, AVRAM Alina, AZZOUZI Kassem, BANKOVSKI Damyan, BARBRY Bernard, BARDOUX Nicolas, BARON Philippe, BAUDET Anne, BAYART Pauline, BAZIN Brice, BEBAHANI Arash, BECQWORT Jean-Pierre, BEN ALI Houssem, BEN SOUSSAN Emmanuel, BENARD Coralie, BENET Vincent, BENGUIGUI Corinne, BENTAL Abdeslam, BENTALEB-BELLATI Sara, BERKELMANS Isabelle, BERNET Jacques, BERNOU Karine, BERTIAUX-VANDAELE Nathalie, BERTOT Pauline, BERTRAND Valérie, BILOUD Emilie, BIRON Nathalie, BISMUTH Benjamin, BLANCHARD Cyril, BLEUEZ Maurice, BLONDEL Fabienne, BLONDIN Valérie, BOBULA Marius, BOHONT Philippe, BOIVIN Eléonore, BON DJEMAH Vanessa, BONIFACE Eric, BONNIERE Philippe, BONVARLET Pierre, BORUCHOWICZ Arnaud, BOSTVIRONNOIS Raoul, BOUALIT Médina, BOUAZZA Ahlem, BOUCHE Bruno, BOUDAILLER Christian, BOURGEAUX Claude, BOURGEOIS-FUMERY Morgane, BOURGUET Arnaud, BOURIENNE Agnès, BOUTALEB Hamza, BOUTHORS Alexis, BRANCHE Julien, BRAZIER Franck, BRIDENNE Marie, BRIHIER Hélène, BRIL Laura, BULOIS Philippe, BURGIERE Pierre, BUTEL Joël, CANVA Jean-Yves, CANVA-DELCAMBRE Valérie, CARDOT Florence, CARETTE Sandrine, CARPENTIER Pierre, CASSAGNOU Michel, CASSAR Jean-François, CASTEX François, CATALA Pascale, CATTAN Stéphane, CATTEAU Sylviane, CAUJOLLE Bernard, CAYRON Gérard, CHANDELIER Catherine, CHARPENTIER Cloé, CHAVANCE-THELU Marthe, CHENY Agathe, CHIRITA Dinu, CHOTEAU Antoine, CLAERBOUT Jean-François, CLERGUE Pierre-Yves, COEVOET Hugues, COHEN Gil, COLIN Marie, COLLET Régis, COLOMBEL Jean-Frédéric, COOPMAN Stéphanie, CORDIEZ Lucie, CORTOT Antoine, CORVISART Jean, COUTTENIER Frédéric, CRINQUETTE Jean-François, CROMBE Valérie, DAOUDI Abdelhakim, DAPVRIL Vincent, DAVION Thierry, DECOSTER Sébastien, DEFONTAINE Laurent, DEGRAVE Nicolas, DEJAEGER Aurélien, DELCENSERIE Richard, DELEPLANQUE Marine, DELESALLE Dorothée, DELETTE Olivier, DELGRANGE Thierry, DELHOUSTAL Laurence, DELMOTTE Jean-Stéphane, DEMMANE Sabri, DEREGNAUCOURT Guy, DESCHEPPER Constance, DESECHALLIERS Jean-Pierre, DESMET Patrick, DESREUMAUX Pierre, DESSEAUX Gérard, DESURMONT Philippe, DEVIENNE Alain, DEVOUGE Eve, DEVROUX Alex, DEWAILLY Arnaud, DHARANCY Sébastien, DI FIORE Aude, DIAZ Emmanuel, DJEDDI Djamal-Dine, DJEDIR Rachid, DOLEH Wissam, DREHER-DUWAT Marie-Laure, DUBOIS Richard, DUBURQUE Clothilde, DUCROT Frédéric, DUCROTTE Philippe, DUFILHO André, DUHAMEL Christian, DUMANT-FOREST Caroline, DUPAS Jean-Louis, DUPONT Frédéric, DURANTON Yves, DURIEZ Arnaud, DUVEAU Nicolas, EL FARISI Mohammadi, EL HACHKAR Khalil, ELIE Caroline, ELIE-LEGRAND Marie-Claire, EOCHE Matthieu, ESSMAEEL Essmaeel, EVRARD Dominique, EVRARD Jean-Paul, FATOME Armelle, FELLAH-SEKKAI Karima, FILOCHE Bernard, FINET Laurent, FLAHAUT Mathilde, FLAMME Camille, FOISSEY David, FOURNIER Peggy, FOUTREIN Philippe, FOUTREIN-COMES Marie-Christine, FRERE Thierry, FUMERY Mathurin, GALAND Julie, GALLAIS Philippe, GAMBLIN Claudine, GANGA Serge, GERARD Romain, GESLIN Guillaume, GHEYSSENS Yves, GHRIB Salah, GILBERT Thierry, GILLET Bénédicte, GODART Denis, GODCHAUX Jean-Michel, GOEGUEBEUR Guetty, GORIA Odile, GOTTRAND Frédéric, GOWER Philippe, GRADOS Lucien, GRANDMAISON Brigitte, GROUX Marion, GUEDON Claire, GUERBEAU Loïc, GUEROULT-DERO Mathilde, GUILLARD Jean-François, GUILLEM Laurence, GUILLEMOT François, GUIMBER Dominique, HADDOUCHE Baya, HAUTEFEUILLE Vincent, HECKETSWEILLER Philippe, HECQUET Geneviève, HEDDE Jean-Pierre, HELLAL Hassina, HENNERESSE Pierre-Emmanuel, HERAUD Michel, HERVE Sophie, HEYMAN Bruno, HOCHAIN Patrick, HOUCKE Philippe, HOUSSIN-BAILLLY Lucie, HUGUENIN Bruno, IOBAGIU Silviu, ISTANBOLI Shata, IVANOVIC Alexsandar, IWANICKI-CARON Isabelle, JANICKI Eric, JARRY Marine, JEAN BART Charlotte, JONAS Claude, JOUGON Julia, JOUVENET Anne, KASSAR Naeim, KATHERIN Fadi, KERLEVEO Alfred, KHACHFE Ali, KIRIAKOS Alfred, KIRIAKOS Jean, KLEIN Olivier, KOHUT Matthieu, KORNHAUSER Richard, KOUTSOMANIS Demetrios, LABERENNE Jean-Eric, LACOTTE Eric, LAFFINEUR Guy, LAGARDE Marine, LAHAYE Anouck, LALANNE Arnaud, LALIEU Ambroise, LANNOY Pierre, LAPCHIN José, LAPRAND Michel, LAUDE Denis, LE COUTEULX Christian, LE GOFFIC Charles, LE GRIX Alain, LE MOUEL Jean-Philippe, LE ROY Pauline, LEBLANC Rachida, LECIEUX Paul, LECLEIRE Stéphane, LECLERC Nathalie, LEDENT Jean, LEFEBVRE Jean, LEFILLIATRE Pascale, LEGRAND Céline, LELONG Patrick, LELUYER Bernard, LEMAITRE Caroline, LEPILEUR Lucie, LEPLAT Antoine, LEPOUTRE-DUJARDIN Elodie, LEPPEUT Gabriel, LEROI Henri, LEROY Maryvonne, LESAGE Benoît, LESAGE Jocelyn, LESAGE Xavier, LESCANNE-DARCHIS Isabelle, LESCUT Dominique, LEURENT Bruno, LEY Delphine, LHERMIE Michel, LIBIER Louise, LISAMBERT Bernard, LOGE Isabelle, LOREAU Julien, LOUVET Alexandre, LOZINGUEZ Joséphine, LUBREZ Henri, LUCIDARME Damien, LUGAND Jean-Jacques, MACAIGNE Olivier, MAETZ Denis, MAILLARD Dominique, MANCHERON Hubert, MANOLACHE Olivia, MARKS-BRUNEL Anne-Bérengère, MARRE Charline, MARTI Raymond, MARZLOFF Eric, MATHURIN Philippe, MAUILLON Jacques, MAUNOURY Vincent, MAUPAS Jean-Luc, MEDAM DJOMO Michèle-Ange, MELCHIOR Chloé, MELKI Ziad, MESNARD B, METAYER Patrice, METHARI Lofti, MEURISSE Franck, MICHAUD Laurent, MODAINE Patricia, MONTHE Angélique, MOREL Loïk, MORIN Mathilde, MORTIER Pierre-Eugène, MORTREUX Perrine, MOUTERDE Olivier, MOZZICONACI Nicolas, MUDRY Jean, NACHURI Maria, NGO Minh Dung, N'GUYEN KHAC Eric, NOTTEGHEM Bertrand, OLLEVIER Vincent, OURAGHI Atika, OUSSADOU Barriza, OUVRY Dominique, PAILLOT Bernard, PAINCHART Claire, PANIEN-CLAUDOT Nicole, PAOLETTI Christian, PAPAZIAN Arsène, PARENT Bruno, PARIS Jean-Claude, PATRIER Philippe, PAUPARD Thierry, PAUWELS Bernard, PAUWELS Mathieu, PETIT Richard, PIAT Muriel, PIOTTE Sandrine, PLANE Christophe, PLOUVIER Bernard, POLLET Eric, POMMELET Pierre, POP Daniela, PORDES Charlotte, POUCHAIN Gérard, PRADES Philippe, PREVOST Jean-Christophe, PRUIT Manon, QUARTIER Gilles, QUEUNIET Anne-Marie, QUINTON Jean-François, RABACHE Alain, RACLOT Gilles, RATAJCZYK Sébastien, REIX Nicole, RENAUT-VANTROYS Thibaud, REVILLION Marine, RIACHI Ghassan, RIAULT Clémentine, RICHARD Nicolas, RICHEZ Cécile, RIMBERT Benoît, ROBINSON Philippe, RODRIGUEZ Juan Daniel, ROGER Jean, ROUX Jean-Marc, RUDELLI Alain, SAINGIER Clémence, SAVOYE Guillaume, SCHLOSSBERG Patrick, SEFRIOUI David, SEGRESTIN Michel, SEGUY David, SEMINUR Célik, SEVENET François, SILVIE Jean, SPYCKERELLE Claire, TALBODEC Nathalie, TAVERNIER Noémie, TCHANDEU Henriette, TECHY Aurore, THELU Jean-Luc, THIEBAULT Henri, THOREL Jean-Marie, THUILLIER Christophe, TIELMAN Guillaume, TODE Manuella, TONNEL Jean, TOUCHAIS Jean-Yves, TOULEMONDE-HUGUET Audrey, TOUMELIN Pierre, TOUZE Yvan, TRAN Léa, TRANVOUEZ Jean-Luc, TRIKI Nadia, TURCK Dominique, TURPIN Justine, VAILLANT Eric, VALMAGE Claude, VANCO Dominique, VANDAELE-BERTIAUX Nathalie, VANDAMME Hélène, VANDER EEKEN Elise, VANDERBERCQ Etienne, VANDERMOLLEN Philippe, VANDEVENNE Philippe, VANDEVILLE Lionel, VANDEWALLE Alain, VANHOOVE Jean-Pierre, VANRENTERGHEM Audrey, VANVEUREN Charlotte, VASIES Iona, VERBIESE Guy, VERLYNDE Juliette, VERMELLE Philippe, VERNE Christine, VERNIER-MASSOUILLE Gwenola, VEZELIER-COCQ Perrine, VIART Juliette, VIGNERON Benoît, VINCENDET Marc, VIOT Jacques, VOIMENT YM, WALLEZ Jean-Yves, WANTIER Michel, WARTEL Faustine, WEBER Jean-Christian, WILLOCQUET Jean-Louis, WIZLA Nathalie, WOLSCHIES Eric, YIMFOR Tajiogue, ZAHARA Oana, ZALAR Alberto, ZAOUI Sonia, ZELLWEGER Anne.
